# Local biodiversity is higher inside than outside terrestrial protected areas worldwide

**DOI:** 10.1038/ncomms12306

**Published:** 2016-07-28

**Authors:** Claudia L. Gray, Samantha L. L. Hill, Tim Newbold, Lawrence N. Hudson, Luca Börger, Sara Contu, Andrew J. Hoskins, Simon Ferrier, Andy Purvis, Jörn P. W. Scharlemann

**Affiliations:** 1 School of Life Sciences, University of Sussex, Falmer, Brighton BN1 9QG, UK; 2 United Nations Environment Programme World Conservation Monitoring Centre, 219 Huntingdon Road, Cambridge CB3 0DL, UK; 3 Department of Life Sciences, Natural History Museum, Cromwell Road, London SW7 5BD, UK; 4 Department of Biosciences, College of Science, Swansea University, Singleton Park, Swansea SA2 8PP, UK; 5 CSIRO Land and Water, Canberra Australian Capital Territory 2601, Australia; 6 Department of Life Sciences, Imperial College, London, Silwood Park, London SL5 7PY, UK

## Abstract

Protected areas are widely considered essential for biodiversity conservation. However, few global studies have demonstrated that protection benefits a broad range of species. Here, using a new global biodiversity database with unprecedented geographic and taxonomic coverage, we compare four biodiversity measures at sites sampled in multiple land uses inside and outside protected areas. Globally, species richness is 10.6% higher and abundance 14.5% higher in samples taken inside protected areas compared with samples taken outside, but neither rarefaction-based richness nor endemicity differ significantly. Importantly, we show that the positive effects of protection are mostly attributable to differences in land use between protected and unprotected sites. Nonetheless, even within some human-dominated land uses, species richness and abundance are higher in protected sites. Our results reinforce the global importance of protected areas but suggest that protection does not consistently benefit species with small ranges or increase the variety of ecological niches.

## Abstract (web summary)

Protected areas are thought essential for biodiversity conservation, but few studies confirm that protection benefits species. Here, Gray and Hill *et al*. analyse a global, taxonomically broad database to show that local species richness and abundance are higher inside protected areas than outside.

## Graphical abstract

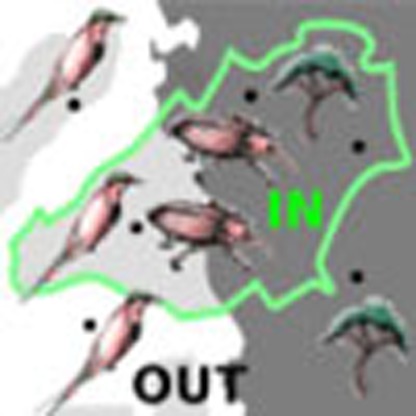

Protected areas are considered an essential strategy for habitat and species conservation^1^. Parties to the Convention on Biological Diversity have committed to increase the terrestrial area currently under protection^2^ from 15.4% to at least 17% in ‘effectively and equitably managed, ecologically representative and well connected' protected areas by 2020 (Aichi biodiversity target 11)^3^. A recent assessment of progress suggests that the coverage target will be met, and that protected area representativeness and management are improving^4^.

However, there is some doubt over the success of protected areas. Management effectiveness reports suggest only 22% of protected areas have ‘sound management'^5^, experts estimate that only half of all tropical reserves are effective^6^ and human pressures are increasing in Latin American, African and Asian protected areas^7^. Declines in animal and plant abundance have been documented inside protected areas^6^,^8^,^9^ and in many countries the effectiveness of protection is being compromised by external pressures and inadequate government support^10^,^11^. Protecting all terrestrial sites of conservation significance could cost US$ 76 billion annually^12^, plus associated opportunity costs (up to US$ 6,500 ha^−1^ for productive agricultural land^13^). Quantifying the effectiveness of protected areas is therefore crucial to justify maintaining and expanding the network.

Evidence from remote sensing suggests that protected areas slow change from ‘natural' to ‘human-modified' land cover^14^ and successfully retain forests^1^. In the tropics, protection reduces deforestation^15^,^16^,^17^, loss of carbon^18^ and forest fires^19^. There is some evidence that changes in land cover and human pressure vary with IUCN Protected Area Management Categories (henceforth ‘IUCN category')^20^, although protected areas that have been assigned categories with more restrictive management objectives have not consistently experienced less land-use change, as their location may be more important than their IUCN category^16^,^17^,^19^,^21^,^22^,^23^,^24^.

Importantly, preventing land-use change does not necessarily conserve species within protected areas (for example, hunting may still occur^25^) and there are few regional or global assessments of how protection affects species and assemblages. Geldmann *et al*.^1^ found that most of the 42 studies in their meta-analysis reported that abundances of species were higher inside protected areas, but acknowledged the limited evidence base. Coetzee *et al*.^22^, reviewing 86 studies, found a positive effect of protection on species richness and abundance. These two meta-analyses are informative but their use of effect sizes—rather than primary data—constrained the biodiversity measures that could be considered. Consequently, it is not yet clear whether protection solely maintains greater numbers of individuals (and therefore higher species richness) or additionally maintains a greater variety of niches. If the latter is true, we expect protection to be associated with more species for a given number of individuals (that is, a higher rarefaction-based richness^26^—hereafter ‘rarefied richness').

A further limitation of some previous studies is that the apparent success of protected areas may be caused by their location rather than protection *per se*^27^. To account for this bias, several analyses of land cover have matched protected locations or pixels to unprotected counterparts having similar values of potential confounding variables such as elevation or slope^14^,^17^,^19^,^28^,^29^,^30^. Alternatively, potential confounding variables have been included as covariates in models of land-use change^31^,^32^, including some analyses that have compared matched pairs of protected and unprotected locations as differences between paired locations remained even after matching^24^. Both previous meta-analyses of protected-area effects on species^1^,^22^ were limited in their ability to account for potential confounding factors using either of these approaches as they lacked geographic coordinates for individual sample sites and data on specific land use types. Therefore, it remains undecided whether protection offers benefits to biodiversity beyond those attributable to reduced land-use change, that is, whether protection can benefit biodiversity even within a particular land use.

Here we assess the effect of protection on species and assemblages using collated primary data rather than effect sizes, quantifying the effects of protection both among and within land uses, while controlling for potentially confounding variables (see Methods). Using the PREDICTS (Projecting Responses of Ecological Diversity In Changing Terrestrial Systems) database^33^, which collated data on species' presence or abundance at sampled sites from peer-reviewed spatial comparisons of different types and intensities of anthropogenic pressures, we calculate four biodiversity measures based on sampled abundances and occurrences at each site (henceforth ‘within-sample' biodiversity measures). The database records geographic coordinates of sampled sites, allowing selection of comparably surveyed sites located inside and outside protected areas by intersecting with the World Database on Protected Areas (WDPA)^34^. We extract data from 156 studies, including 13,669 species of vertebrates, invertebrates and plants, that had sites both inside (*n*=1,939 sites) and outside (*n*=4,592 sites) 359 terrestrial protected areas (Fig. 1) and use mixed-effects models to assess the effects of protection while accounting for among-study differences in sampling methodology. Although this represents a small fraction of the protected area network (0.18%), it is substantially larger than previous meta-analyses^1^,^22^ and spans 48 countries, 101 ecoregions and 13 of the 14 terrestrial biomes. The sampled protected areas show a similar distribution to that of all terrestrial protected areas in the WDPA^34^ in terms of size, year of establishment and IUCN category and are reasonably representative in terms of how their total area is divided among land uses, ecoregions and biomes (Supplementary Fig. 1). We find that within-sample species richness and total abundance are significantly higher at sites inside than outside protected areas. Importantly, significant interactions between protection and land use in our models indicate that protection does more than merely prevent land-use change (suggesting protection benefits biodiversity even within human-dominated land uses). However, in contrast to our expectations, protection has no effect on either rarefied richness (suggesting protection has little effect on the number of species present for a given number of individuals, and therefore does not increase the variety of viable niches available) or endemicity (suggesting protection has little effect on the proportion of individuals within a community that have narrow geographic ranges). Finally, we estimate that on average the global protected area network is 41% (95% confidence interval (CI): 2.0 to 81%) effective at retaining within-sample species richness and 54% (95% CI: 0 to 136%) effective at retaining local abundance.

## Results

### Local biodiversity inside and outside protected areas

**Figure 1:**
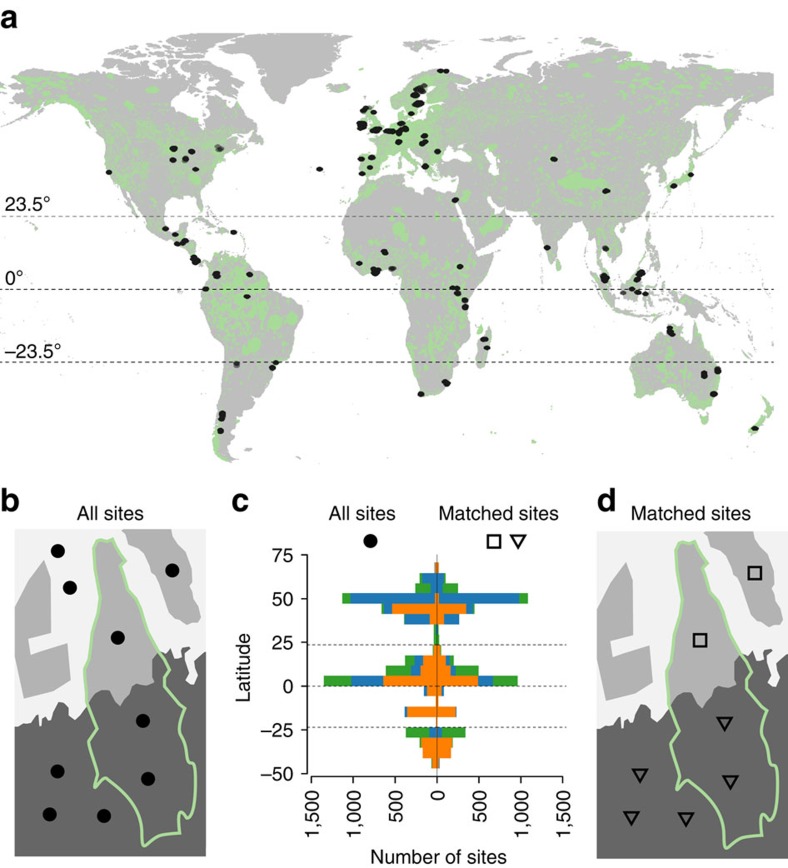
Spatial and taxonomic structure of data. (**a**) Location of 4,592 sites outside and 1,939 inside 359 protected areas (pale green; all terrestrial protected areas from ref. 34) from 156 studies in the full (all sites) dataset; horizontal lines indicate equator and tropics of Cancer and Capricorn; world map from ref. 51. (**b**) Diagram of a single study, showing sampling sites inside and outside a protected area (green outline) and different land uses (shades of grey). (**c**) Latitudinal and taxonomic distribution of sites by 6° latitudinal bands for all sites (left) and subset of sites matched by land use (right); colours correspond to taxonomic groups (green=plants, blue=invertebrates, orange=vertebrates). (**d**) Diagram of the same study as (**b**) showing subsets of sites matched by land use (matched-sites data; this included 144 studies with 3,296 sites outside and 1,719 inside 313 protected areas). Sampled protected areas have similar distribution to all terrestrial protected areas in terms of size, year of establishment and IUCN Protected Areas Management Category (henceforth IUCN category) and broadly similar proportion of total area in each of the land uses, ecoregions and biomes represented (Supplementary Fig. 1).

**Figure 2:**
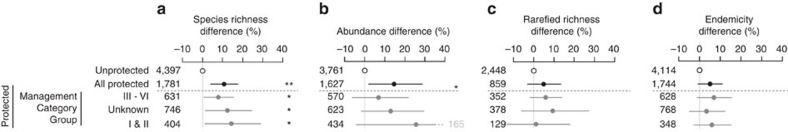
Effects of terrestrial protected areas on four local biodiversity measures. (**a**) Species richness, (**b**) total abundance, (**c**) rarefied richness and (**d**) endemicity at sites inside (filled circles) relative to sites outside protected areas (open). Estimates are given separately for protected areas in different management category groups (grey circles; least restrictive (IUCN categories III–VI), unknown (missing IUCN category, potentially mixed set of categories) and most restrictive land management regimes (IUCN categories I and II)). Bars indicate 95% CIs; **P<0.01 and *P<0.05. Number of sampled sites in each category is shown; sample sizes vary between panels due to differences in the use of occurrence and abundance data to calculate biodiversity measures (see Supplementary Information). Separate generalized linear mixed effects models were run for each response variable (see Supplementary Information for further information). Supplementary Fig. 3 gives the corresponding results for analyses where sites were matched across the protected area boundary by land use.

Samples from protected sites (Fig. 1b) contained 10.6% more species (95% CI: 4.1 to 17.6%; *χ*^2^=9.99, *df*=1, *P=*0.002; Fig. 2a) and 14.5% more individuals (95% CI: 2.0 to 28.7%; *χ*^2^=5.09, *df*=1, *P=*0.024; Fig. 2b) than samples from unprotected sites. If protection maintains a wider set of viable niches, we would also expect rarefied richness (that is, the number of species expected if each site within a study had yielded the same number of individuals) to be higher inside protected areas. However, there was no significant effect of protection on rarefied richness (95% CI: −31.06 to 13.5%; *χ*^2^=1.33, *df*=1, *P*>0.2; Fig. 2c and Supplementary Table 1), suggesting that the higher within-sample species richness of protected sites largely reflects higher overall abundance (as the number of species detected increases with the number of individuals sampled^26^). Assemblages in protected areas might also be expected to have a higher proportion of endemic species and a smaller proportion of widespread species. However, we found the effect of protection on endemicity (a measure we calculated as the inverse of community-weighted mean geographic range size) was marginally nonsignificant (95% CI: −0.6 to 11.1%; *χ*^2^=2.99, *df*=1, *P=*0.08; Fig. 2d).

Analyses of spatial comparisons cannot reveal the mechanism behind differences between protected and unprotected sites. Protected areas may have been selected based on pre-existing biodiversity gradients, or protected area management may result in the preservation of populations lost from surrounding areas. These mechanisms are not mutually exclusive. Time-series data on species and assemblages inside and outside protected areas would be helpful for determining their relative importance. Additionally, we may underestimate the overall contribution of protection, as many protected areas aim to protect biodiversity features not included in our analyses, such as beta diversity, particular rare species, migratory routes or ecological processes^20^.

Protected areas with more restrictive management objectives are expected to retain more biodiversity. Although IUCN categories are not necessarily applied consistently across countries^20^, several studies have used them to compare biodiversity across management objectives^16^,^17^,^19^,^21^,^22^,^23^. As most of these studies focus on land cover (particularly deforestation), there is little information on how effects on species differ among protected areas in different IUCN categories; the limited available evidence indicates a positive effect size for both the most restrictive IUCN categories and for one category that allows some extraction^22^. We found no significant differences among protected area management category groups (three groups, in order of lowest to highest restriction on human activity: IUCN category III–VI, IUCN category unknown (a mixture of categories, considered an intermediate category on average) and IUCN category I and II; Fig. 2a; all comparisons among protected groups gave *P*>0.2), but samples from protected sites in each management category group had higher species richness than those from unprotected sites (Fig. 2a; *χ*^2^=11.18, *df*=3, *P=*0.011. There was large variation but no significant difference in abundance, rarefied richness or endemicity among management category groups and unprotected sites (Fig. 2 and Supplementary Table 1). The heterogeneity within groups could reflect conservation objectives not captured in a protected area's IUCN category^35^ or differences in how these categories have been applied^20^.

### Effects of protection within and among land uses

As all analyses above accounted for differences in elevation, slope and agricultural suitability, differences in land use are the most likely explanation for higher species richness and total abundance in samples from protected sites. We used two approaches to test this hypothesis. First, we included site-specific land use as a fixed effect in our models; and second, we restricted our dataset to sites matched by land use across the protected area boundary (Fig. 1d). In both cases, we also explored whether the effect of protection varied with latitudinal zone and taxonomic group.

### Land use and the effects of protection

**Figure 3:**
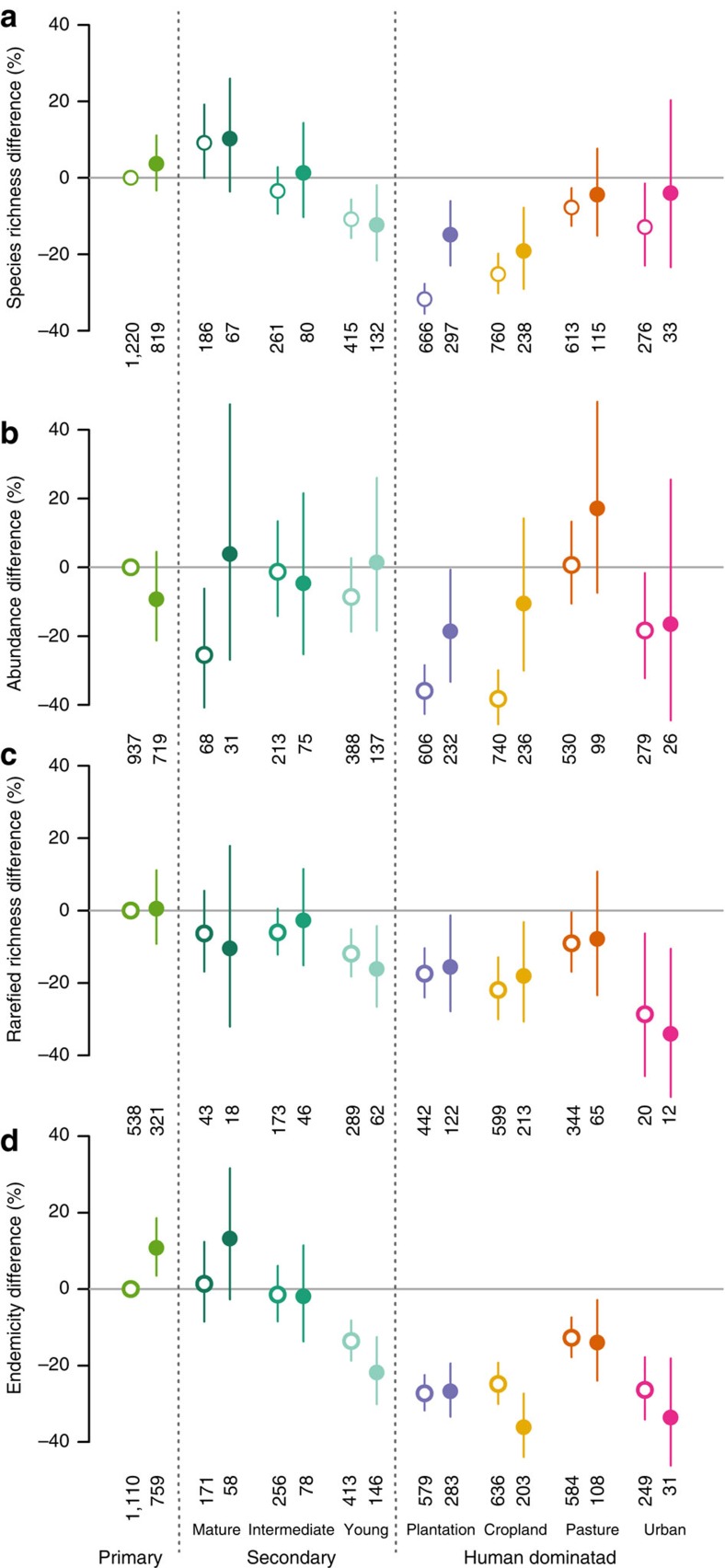
Effects of protection on four biodiversity measures across eight land use types. (**a**–**d**) Sites outside (open circles) and inside (filled circles) protected areas in different land uses (colours: from left to right: primary vegetation; mature, intermediate and young secondary vegetation; plantation; cropland; pasture; urban). Error bars show 95% CIs. The number of sites in each type of land use and protection is given underneath each data point. Separate generalized linear mixed effects models were run for each response variable (see Supplementary Information for further information). Supplementary Fig. 2 gives corresponding analyses for taxonomic group, latitudinal zone and use intensity.

The effect of protection on within-sample species richness, abundance and endemicity varied among land uses (Fig. 3; *χ*^2^=15.26, *df*=7, *P*=0.033; *χ*^2^=19.12, *df*=7, *P=*0.008; *χ*^2^=25.05, *df*=7, *P=*0.001, respectively) but again the effect on rarefied richness did not (Fig. 3c and Supplementary Table 3). The last result reinforces the finding that samples from protected sites do not have more species for a given number of individuals, even across land uses. Protection had little effect on biodiversity measures at sites within primary and secondary vegetation, probably because such sites tend to experience limited human pressure whether outside or inside protected areas. However, in human-dominated land uses—particularly plantation and cropland—samples from protected sites contained significantly more individuals and species than those from unprotected sites (Fig. 3a,b), but only in the tropics (Supplementary Table 2, Supplementary Fig. 2e and f). The greater effect within the tropics is encouraging given they are often considered a high conservation priority (for example, ref. 36). However, given recent acceleration in human activity in the tropics^37^, tropical landscapes may be experiencing an extinction debt that has already been repaid in temperate protected areas. Perhaps surprisingly, endemicity was lower at protected than unprotected sites in human-dominated land uses, particularly in cropland (Fig. 3d and Supplementary Table 2) and for vertebrates and plants (Supplementary Table 2 and Supplementary Fig. 2d). This effect suggests that protected areas in human-dominated land uses may either benefit species with wide ranges or may have been located specifically to protect them (for example, migratory birds).

If protection ameliorates human pressures even within land uses, differences in land-use intensity could explain the higher biodiversity at protected than unprotected sites within the same land-use type. The PREDICTS database specifies three levels of use intensity within each land-use type^33^: minimal (for example, very limited levels of disturbance for natural land uses, low-intensity agriculture), light (for example, some extraction of timber, hunting or pesticide application) and intense (for example, clear-felling, high level of hunting, intensive agriculture, highly urbanized). Even when comparing unprotected and protected sites experiencing the same use intensity in human-dominated land uses, samples from protected sites consistently contained more individuals and species than those from unprotected sites (Supplementary Table 2 and Supplementary Fig. 2i,j). These results suggest an influence of factors not captured in our measure of use intensity (for example, the condition of the wider landscape, or habitat restoration).

### Effect of protection when sites are matched by land use

**Figure 4:**
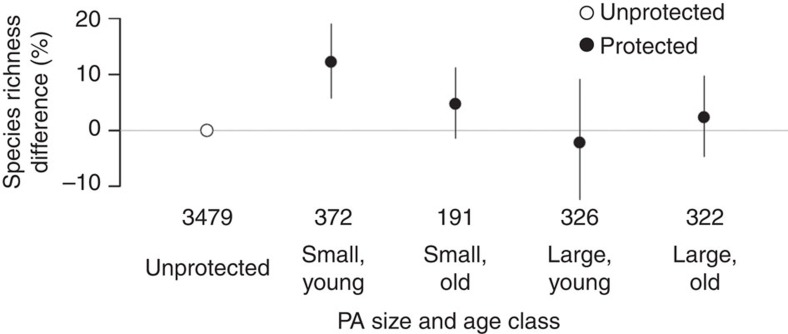
Effects of protected area age and size class on within-sample species richness. The number of sites sampled outside and inside protected areas is given for each age and size combination. Effects were tested using a generalized linear mixed effects model with a Poisson error distribution (log link). See also Supplementary Fig. 4.

Analysing only the sites within each study for which land use could be matched across the protected area boundary (Fig. 1d), we found no significant effect of protection on any biodiversity measure for any management category group, taxonomic group or latitudinal zone (Supplementary Table 3 and Supplementary Fig. 4). However, samples from protected areas that were both young (<20 years) and small (<400 km^2^) had higher species richness than samples from unprotected sites (Fig. 4; *χ*^2^=16.22, *df*=4, *P=*0.003); rarefied richness again did not differ significantly (Supplementary Table 3). It is possible that more recently designated protected areas have targeted areas of high species richness more precisely through use of spatial prioritization algorithms (for example, ref. 38), or that older protected areas have declined in species richness. The effect of protected area size/age class on abundance and endemicity also varied among taxonomic groups (*χ*^2^=25.38, *df*=8, *P=*0.001; *χ*^2^=16.64, *df*=8, *P=*0.034, respectively, Supplementary Fig. 4), with higher invertebrate abundance, vertebrate abundance and plant endemicity in samples from larger, older protected areas. These results suggest that larger, older protected areas may have been designated to protect, or have retained or increased the local abundance of animals and less geographically widespread plants.

### Estimating the global effectiveness of protected areas

We extrapolated from our models of local biodiversity inside and outside protected areas (Fig. 3) to estimate the effectiveness of the current global protected area network at retaining site-level biodiversity. Our measure of effectiveness would be 0% if sites within protected areas are, on average, as diverse as unprotected sites, and 100% if the biodiversity of protected sites is, on average, as high as for ‘pristine' sites (minimally-impacted primary vegetation; note that the scale is not bounded at either 0% or 100%). On this scale, we estimate that on average the global protected area network is 41% (95% CI: 2 to 81%) effective at retaining within-sample species richness and 54% (95% CI: 0 to 136%) effective at retaining local abundance.

### How to improve the global protected area network

An important question for environmental decision makers is whether biodiversity will benefit more from increasing restrictions on human activity in existing protected areas or from expanding the network^39^,^40^. Although the trend towards higher species richness and abundance at sites in protected areas with more restrictive management objectives was not statistically significant, the coefficients suggest the effects of management restrictiveness may be large (Fig. 2a,b). The large uncertainty seen in these models reflects both a lack of precise data on the objectives of protected area management and on effectiveness, and a lower number of sites with the most restrictive management. Better information on protected area management intent and effectiveness is needed to confidently quantify the biodiversity outcome of increasing management restrictions. However, to demonstrate the importance of having improved information on management, we used our model coefficients (Fig. 2a,b) to estimate that the effectiveness of the protected area network could be increased to 94% (95% CI: 50 to 139%) for average within-sample species richness and 167% (95% CI: 0 to 392%) for average within-sample abundance if all protected areas enforced the most restrictive management objectives (that is, IUCN categories I or II). To raise average within-sample species richness worldwide by the same amount solely through expanding the current protected area network (i.e., with the existing distribution among IUCN categories) would require protection of 22% (95% CI: 12 to 63%) of terrestrial area; for within-sample abundance, the corresponding figure is 31% (95% CI: 13 to 299%). The wide CIs on these estimates highlight the urgent need for improved data on the management of protected areas. To help decide whether to expand or change the management of the protected area network requires information on the costs of expansion versus changes in management, the representation of species not currently under protection^41^ and the extent to which these options achieve other globally agreed conservation targets^42^. Nevertheless, other recent studies also suggest that increasing the performance, rather than the total coverage of protected areas, may achieve the desired outcomes for biodiversity more efficiently^39^,^40^,^43^,^44^: this is an important issue requiring further study.

## Discussion

In summary, these first detailed global analyses of site-level data on a large, taxonomically broad set of species show that, overall, samples from protected sites contained more individuals and species than samples from unprotected sites. By contrast, protected sites did not consistently have higher rarefied richness or levels of endemicity—both measures of community characteristics that are often considered when setting conservation priorities. The greatest differences in species richness and abundance occurred across land uses: protected areas are most effective where they minimize human-dominated land use, especially where they safeguard primary or mature secondary vegetation. However, the positive effect of protection within human-dominated land uses, particularly in the tropics, shows that land use is not the only cause of higher biodiversity within protected areas. Better data on management is needed to quantify biodiversity benefits of restricting human activity inside protected areas but, if the trend in our data is confirmed, more restrictive protected area management across the current network could be as important as extending the network. Importantly, we cannot discern whether protection has prevented losses in site-level biodiversity seen in surrounding areas, increased numbers of individuals, or retained a pre-existing biodiversity gradient. Nonetheless, these analyses represent a substantial advance in knowledge about several measures of biodiversity inside versus outside protected areas. Our results reinforce recent calls^45^ for increased support and recognition of the importance of protected areas worldwide^10^,^11^, but highlight that the network is not currently effective for all measures of local biodiversity.

## Methods

### Data

For each sampling location or site in the PREDICTS database (November 2014 version), we calculated within-sample species richness, total abundance of individuals, rarefied richness (based on the fewest individuals at any site within each study) and community weighted mean log_10_ geographic range size—the inverse of which was then plotted to give our endemicity measure. Each species' range size was derived from its global occurrence in the Global Biodiversity Information Facility database. We recognise biases in the Global Biodiversity Information Facility data, but these are mitigated to some extent by our hierarchical modelling approach and our estimates compare reasonably well with estimates based on other data sources, listed in full in the Supplementary Information. Land use was classified using the study authors' description for each site; this method has been shown to be repeatable^33^. Sites were considered to be protected if their geographical coordinates fell inside protected areas from the World Database on Protected Areas^34^ (see Supplementary Methods). We then derived two datasets: the first included all studies with sites inside and outside protected areas (all-sites data; Fig. 1b); the second retained only those sites from each study for which land use could be matched across the protected area boundary (matched-sites data 2; Fig. 1d). All sources of biodiversity data are listed in the Supplementary Information.

### Analyses

We used generalized linear mixed-effects models to account for differences in response variables due to study-specific methodologies and the spatial structure of sites^46^. The PREDICTS data present a rare opportunity to compare sites inside and outside protected areas, but do not have the geographic coverage required for a stricter counterfactual approach^14^,^17^ in which sites are individually matched. To reduce the risk that any differences observed between sites inside and outside protected areas were caused by biases in the location of protected areas^27^, we considered elevation^47^ and derived slope at c. 1 km^2^ resolution and agricultural suitability^48^ at 10 km^2^ resolution as covariates in all models (see Supplementary Information for further details). To ensure independence of all variables in the model, we intentionally included only these three confounding variables that we considered to be fully independent of the presence of a protected area. For example, distances to roads and markets are affected by the presence of protected areas so are not independent confounding factors (see Supplementary Information for details). We sequentially compared models with and without each fixed effect and at each step dropped the term with the highest *P*-value, until all terms had *P*<0.05 (ref. 49).

### Assessing protection effects

We tested for biodiversity differences between sites inside and outside protected areas using the all-sites data, treating protection status (inside vs outside a protected area) as a fixed effect. We then tested whether biodiversity measures differed between management category groups by re-coding IUCN category as a four-level factor: unprotected, IUCN category III–VI, IUCN category unknown, and IUCN category I and II.

### Assessing protection effects within and among land uses

We used two approaches to test whether biodiversity differences between protected and unprotected sites varied with land use. First, using the all-sites data, we modelled the response of each biodiversity measure to protection status, land use, and their interaction. We also tested for the three-way interaction between land use, protection and either use intensity, latitudinal zone or taxonomic group. Second, using the matched-sites data, we re-ran models with protection status, and then with management category group as a fixed effect. We also split the matched-sites data by latitudinal zone and taxonomic groups to assess whether these factors influenced the effect of protection. Finally, we tested whether the site-level biodiversity response to protection varied with the size/age class of the protected area [four-level factor with all combinations of young (<20 years), old (20–85 years), small (<400 km^2^) and large (400–12,000 km^2^); these thresholds between categories were selected to give a similar number of sites in each group].

### Estimating global protected area effectiveness

The global effectiveness of protected areas (*e*) was estimated from *e*=1−(1−*i*)/(1−*o*), where modelled site-level biodiversity inside (*i*) and outside (*o*) protected areas are expressed as a proportion of that under ‘pristine' conditions. We calculated the ratio of *i*/*o* from the model estimates for biodiversity inside relative to outside protected areas in each land use (Fig. 3), where each land-use parameter was weighted by the proportion of global terrestrial area within that land-use type. This value of *i*/*o* could then be used to solve an equation expressing the global state of site-level biodiversity: 1−*r*=*ai*+(1−*a*)*o*, where *r* is the estimated global average loss of site-level biodiversity relative to pristine^46^ and *a* is the fraction of the total land area that is protected^50^. Solving this equation for *i* and *o* allowed us to estimate *e*. Finally, by using estimates for the effect of protection in IUCN categories I and II (Fig. 2a,b) to give *i*/*o*, we estimated *e* under the more restrictive management scenario. By rearranging the equations we estimated the total protected area (*a*) needed to obtain the same average local biodiversity outcome (1−*r*) inferred under this more restrictive management scenario. See Supplementary Information for more details.

### Data availability

The biodiversity data that support the findings of this study are available in the Natural History Museum data portal (data.nhm.ac.uk) with the identifier dx.doi.org/10.5519/0095544. R scripts are available at http://github.com/claudialouisegray/PREDICTS_WDPA.

## Additional information

**How to cite this article:** Gray, C. L. *et al*. Local biodiversity is higher inside than outside terrestrial protected areas worldwide. *Nat. Commun.* 7:12306 doi: 10.1038/ncomms12306 (2016).

## Supplementary Material

Supplementary Information Supplementary Figures 1-4, Supplementary Tables 1-3, Supplementary Note 1, Supplementary Methods and Supplementary References.
