# Supplementary material for: Local biodiversity is higher inside than outside terrestrial protected areas worldwide
Source: Nat Commun. 2016 Jul 28;7:12306. doi: 10.1038/ncomms12306 (PMC4974472; doi:10.1038/ncomms12306)
Supplement: Supplementary Information — Supplementary Figures 1-4, Supplementary Tables 1-3, Supplementary Note 1, Supplementary Methods and Supplementary References. [file ncomms12306-s1.pdf]

Supplementary Figures

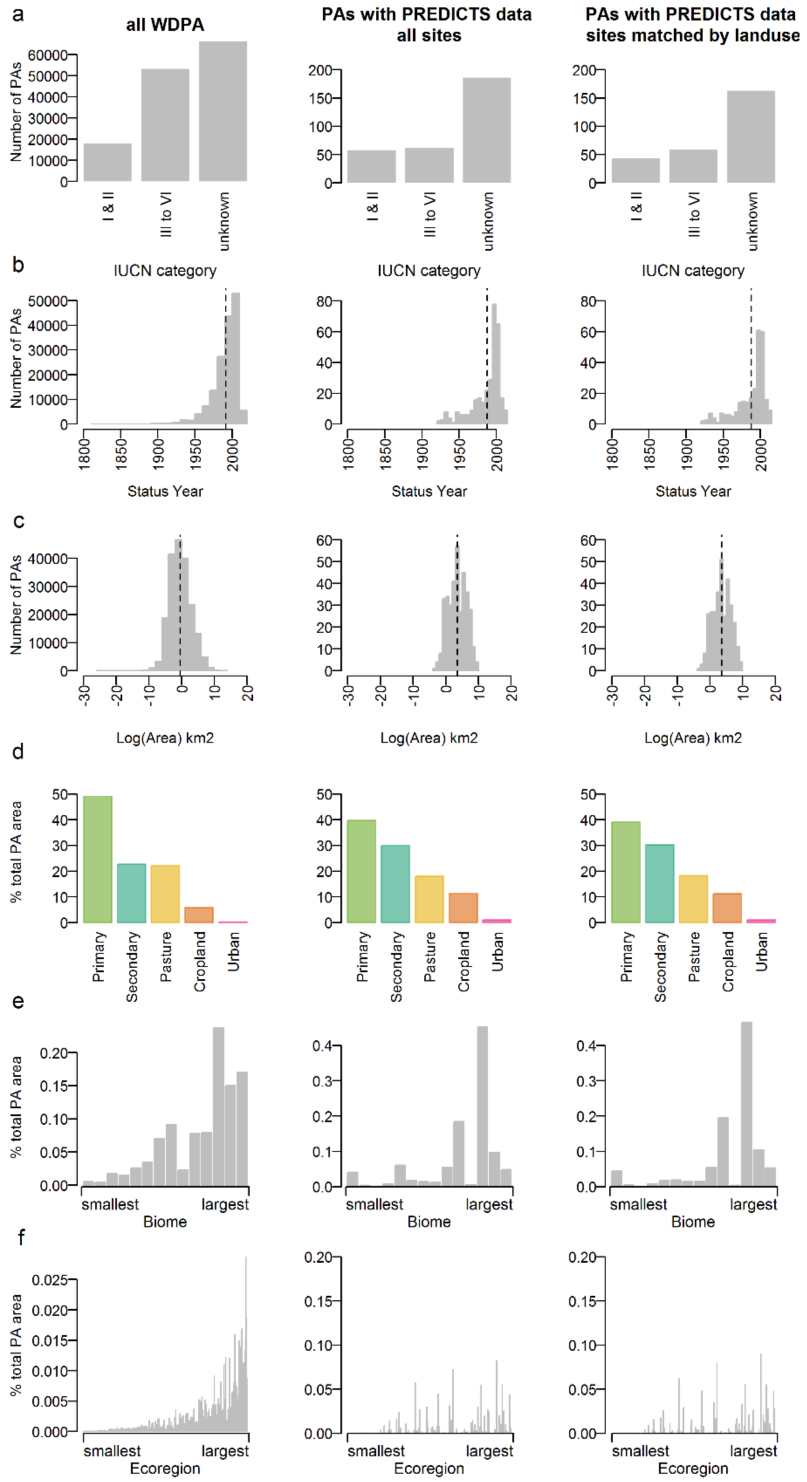

Supplementary Figure 1 | Overview of protected area data. IUCN protected area management category (**a**), year of establishment (status year) (**b**), size (**c**), and proportion of total land area within each of 5 land use types (**d**), biomes (**e**) and ecoregions (**f**) for the World Database on Protected Areas[1] (July 2014 version, n = 194,598; left column), all protected areas (PAs) with data in this study (n = 359; middle column) and the PAs for which sites could be matched by land use within the same study (n = 313; right column). Dashed lines show mean values.

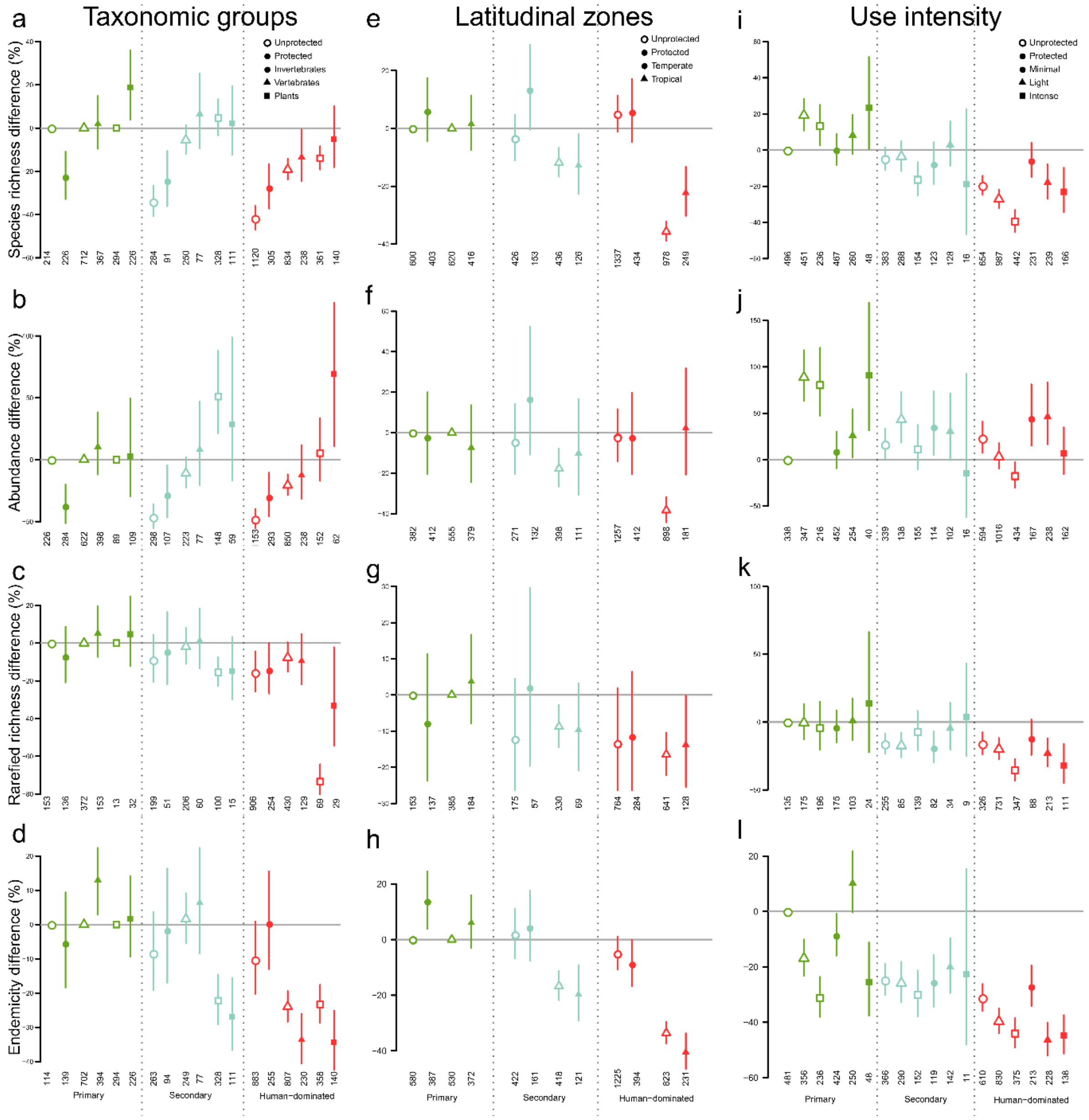

Supplementary Figure 2 | Effects of land use types on biodiversity measures. Effects of three land use types (primary [green], secondary [blue] and human dominated [red]) and protection (unprotected = open, protected = filled symbols) on species richness (**a**), abundance (**b**), rarefied richness (**c**) and endemicity (**d**) of invertebrates (circles), vertebrates (triangles) and plants (squares), in temperate (circles) and tropical (triangles) zones (**e – h**) and for three levels of use intensity: minimal (circles), light (triangles), and intense (squares) (**i – l**). Error bars show 95% confidence intervals. The number of sites in each land use type and protection status is given underneath the corresponding data point.

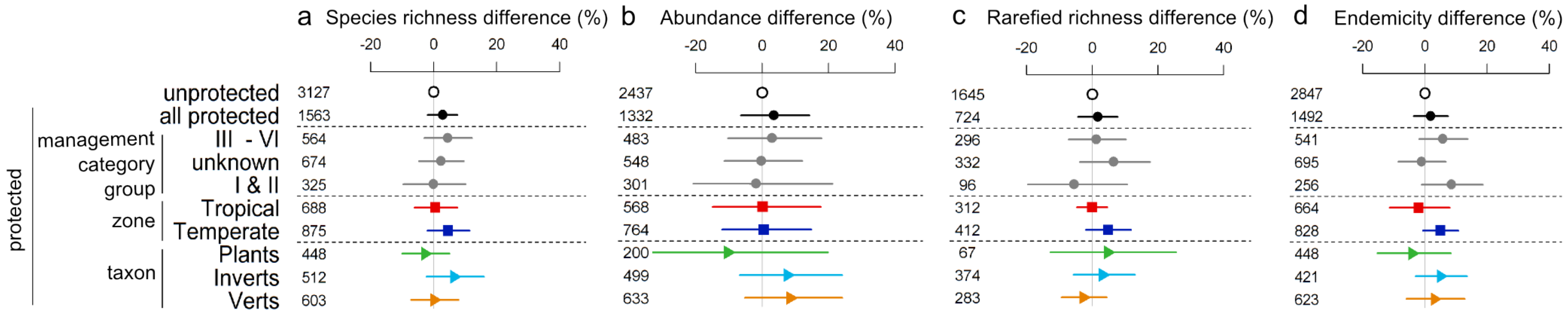

Supplementary Figure 3 | Effects of terrestrial protected areas on four biodiversity measures. Effects of terrestrial protected areas on four local biodiversity measures when data from each study is restricted to sites in the same land use. Response of species richness (**a**), total abundance (**b**), rarefied richness (**c**), and endemicity (**d**) to protection (black circles) relative to unprotected (open). Estimates are given separately for protected areas with different management regimes (grey circles; the least restrictive [IUCN categories III-VI], unknown [missing IUCN category, potentially a mixture of categories], and the most restrictive land management regimes [IUCN categories I & II]); for tropical (red squares) and temperate zones (blue squares) and for different taxonomic groups (triangles). Error bars show 95% confidence intervals. Sample sizes (number of sites) shown.

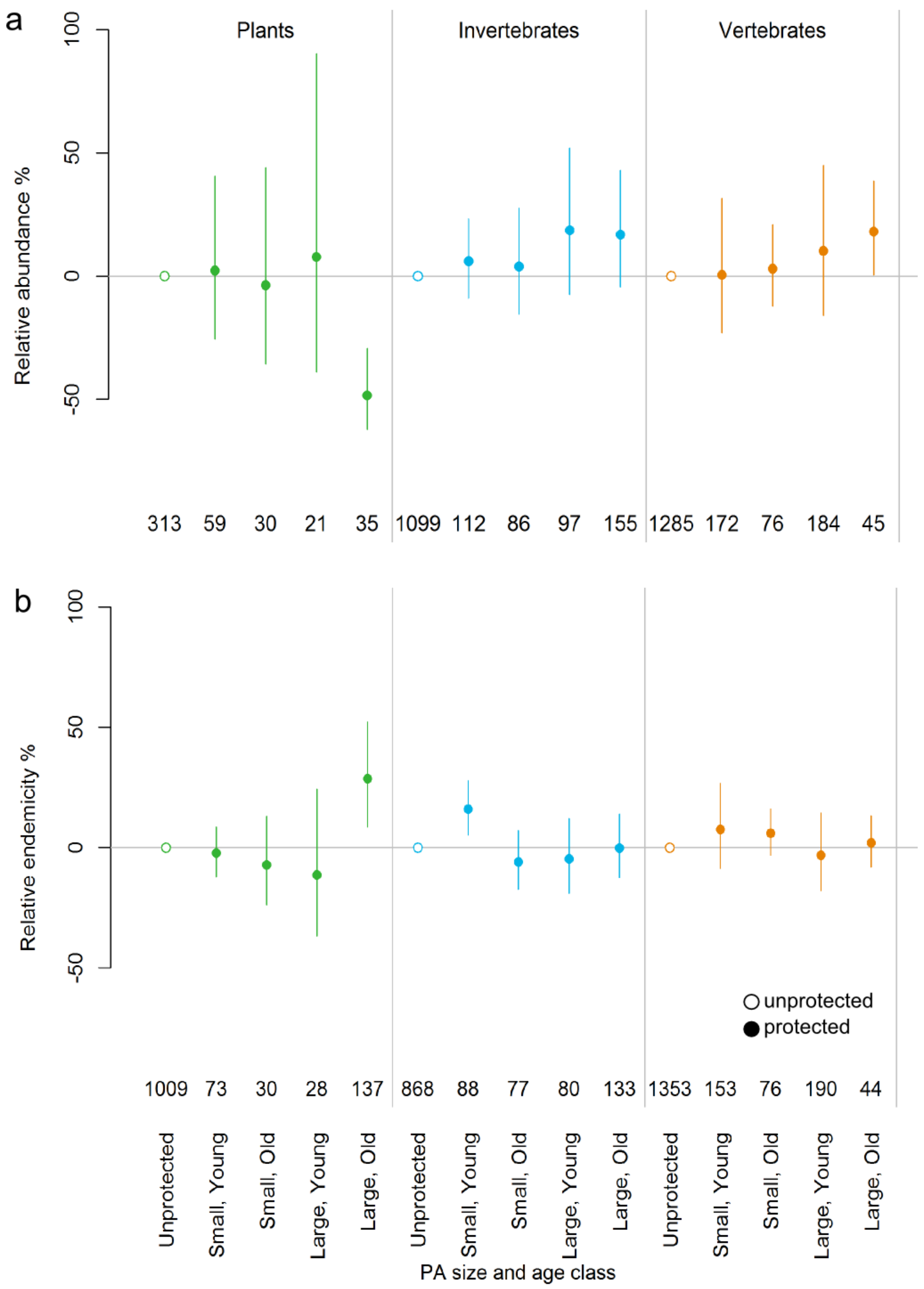

Supplementary Figure 4 | Effects of protected area age and size on abundance and endemicity. Effects of protected area age and size class on abundance (**a**) and endemicity (**b**) for three broad taxonomic groups, plants (green), invertebrates (blue) and vertebrates (orange). The number of sites in each age and size combination is given underneath each data point, with unprotected sites (open) and protected (filled triangles).

Supplementary Tables

Supplementary Table 1 | Statistics from the minimum adequate models for which the results are presented in Fig. 2. The first column specifies the data subset used in the model. The datasets “>80% known range”, “All data rar. richness”, “No small studies” and “No PA focus” refer to the four sensitivity analyses. In these cases we report only the results for the term of interest, not the confounding variables. χ^2, df and p-values are given for the comparison of the model with and without the term specified in the second column. Protection was a two-level factor specifying whether each site was protected or unprotected, management category group was a four-level factor specifying whether each site was: unprotected, in a protected area with IUCN category I & II, IUCN category III to VI, or in a protected area with IUCN category unknown. Agricultural suitability, slope and elevation were all continuous variables, for which cubic, quadratic and linear terms were tested sequentially. The polynomial term (poly) retained in the final model is given for the continuous variables. dAIC gives the change in AIC from a model with the term specified to one without.

		**Species richness**					**Abundance**					**Rarefied richness**					**Endemicity**				
**Dataset**	**Term removed**	**poly**	χ^2	**df**	**p**	**dAIC**	**poly**	χ^2	**df**	**p**	**dAIC**	**poly**	χ^2	**df**	**p**	**dAIC**	**poly**	χ^2	**df**	**p**	**dAIC**
All-sites	protection		9.99	1	0.002	7.99		5.09	1	0.024	3.09		1.33	1	0.248	-0.67		2.99	1	0.084	0.99
	slope	1	0.40	1	0.529	-1.60	1	0.17	1	0.681	-1.83	1	0.06	1	0.813	-1.94	3	6.73	1	0.009	4.73
	elevation	1	46.90	1	<0.001	44.90	1	0.76	1	0.382	-1.24	3	9.15	1	0.002	7.15	2	35.14	1	<0.001	33.14
	ag.suitability	1	7.82	1	0.005	5.82	1	1.58	1	0.209	-0.42	3	10.17	1	0.001	8.17	3	9.8/9	1	0.002	7.89
All-sites	management category		11.18	3	0.011	5.18		5.04	3	0.169	-0.96		3.21	3	0.360	-2.79		3.67	3	0.299	-2.33
	slope	1	0.58	1	0.448	-1.42	1	0.04	1	0.847	-1.96	1	0.09	1	0.764	-1.91	3	5.90	1	0.015	3.90
	elevation	2	4.32	1	0.038	2.31	1	1.21	1	0.271	-0.79	1	4.01	1	0.022	3.22	2	36.78	1	<0.001	34.78
	ag.suitability	1	8.51	1	0.004	6.51	1	1.81	1	0.178	-0.19	1	11.42	1	<0.001	9.43	3	7.11	1	0.008	5.11
>80% known range	protection																	1.19	1	0.275	-0.81
	management category																	4.38	3	0.223	-1.62
All data rar. richness	protection		4.41	1	0.036	2.41															
	management category		7.03	3	0.071	1.03															
No small studies	protection		7.95	1	0.005	5.95		6.34	1	0.012	4.34		1.58	1	0.208	-0.41		2.96	1	0.085	0.96
	management category		8.68	3	0.034	2.68		5.44	3	0.142	-0.55		3.21	3	0.360	-2.79		5.00	3	0.172	-0.10
No PA focus	protection		6.43	1	0.011	4.44		3.91	1	0.048	1.91		0.88	1	0.349	-1.12		1.74	1	0.187	-0.26
	management category		8.21	3	0.042	2.21		4.27	3	0.234	-1.73		3.30	3	0.347	-2.70		2.16	3	0.541	-3.84

Supplementary Table 2 | Statistics from mixed effects models testing for effect of land use and protection, using all data from studies with at least one site inside and outside a protected area. The first column specifies the data used. The datasets “>80% known range”, “All data rar. richness”, “No small studies” and “No PA focus” refer to the four sensitivity analyses. In these cases we report only the results for the term of interest, not the confounding variables. χ^2, df and p-values are given for the comparison of the model with and without the term specified. Protection was a two level factor specifying whether each site was protected or unprotected, LU8 was an eight level factor describing the land use of each site (primary, mature secondary, intermediate secondary, young secondary, plantation, cropland, pasture, urban). LU3 was a three level factor grouping each level from land use (8) into one of three coarser categories: primary, secondary or human dominated land use. Use intensity was a three level factor (intense, medium and light use). Taxon was a three level factor (plants, vertebrates and invertebrates) and latitudinal zone was a two level factor (tropical and temperate). LUPA refers to the compound variable used to express the land use (3):protection interaction, with six levels, one for each possible combination of protection and land use (3). LUUI refers to the compound variable used to express the land use (3):use intensity interaction, with nine levels, one for each possible combination of land use (3) and use intensity. Agricultural suitability, slope and elevation were all continuous variables (for which cubic, quadratic and linear terms were tested sequentially) with “poly” giving the polynomial term retained in the final model. Where the term was not significant, χ^2, df and p-value are given for the linear term only. dAIC gives the change in AIC from a model with the term specified to one without.

		Species richness					Abundance					Rarefied richness					Endemicity				
**Dataset**	**Term removed**	**poly**	χ^2	**df**	**p**	**dAIC**	**poly**	χ^2	**df**	**p**	**dAIC**	**poly**	χ^2	**df**	**p**	**dAIC**	**poly**	χ^2	**df**	**p**	**dAIC**
All-sites	LU8		246.49	7	<0.001	232.49		103.58	7	<0.001	89.58		61.84	7	<0.001	47.84		298.61	7	<0.001	284.61
	protection		4.80	1	0.028	2.80		2.58	1	0.11	0.58		0.54	1	0.464	-1.46		0.17	1	0.681	-1.83
	slope	1	0.06	1	0.814	-1.94	1	0.06	1	0.799	-1.94	1	0.72	1	0.395	-1.28	3	11.29	3	0.010	5.29
	elevation	3	314.29	3	<0.001	25.29	1	2.77	1	0.096	0.77	3	13.21	3	0.004	7.21	3	105.48	3	<0.001	99.48
	ag.suitability	3	10.78	3	0.013	4.78	1	1.87	1	0.171	-0.13	3	33.18	3	<0.001	27.16	3	11.53	3	0.009	54.53
	taxon		10.10	2	0.006	6.10		21.51	2	<0.001	17.51		4.21	2	0.122	0.21		45.39	2	<0.001	41.39
	latitudinal zone		5.29	1	0.02	3.29		4.44	1	0.035	2.44		6.32	1	0.012	4.32		63.69	1	<0.001	61.69
	LU8:protection		15.26	7	0.033	1.26		19.12	7	0.008	54.12		1.74	7	0.973	-12.26		25.05	7	0.001	11.05
>80% known range	LU8: protection																	25.38	7	<0.001	11.38
All data rar. richness	LU8: protection		15.64	7	0.029	1.64															
No small studies	LU8: protection		17.29	7	0.016	3.29		18.79	7	0.009	4.79		4.10	7	0.770	-9.90		32.45	7	<0.001	18.45
No PA focus	LU8: protection		23.08	7	0.002	9.08		16.61	7	0.020	2.61		4.51	7	0.719	-9.49		20.73	7	0.004	6.73
All-sites	LU3		243.38	2	<0.001	239.40		58.80	2	<0.001	54.80		48.52	2	<0.001	44.52		238.22	2	<0.001	234.22
	protection		3.63	1	0.056	1.64		2.44	1	0.118	0.44		0.65	1	0.418	-1.35		0.36	1	0.550	-1.64
	slope	1	0.03	1	0.871	-1.97	1	0.00	1	0.995	-2.00	1	0.76	1	0.383	-1.24	3	10.50	3	0.015	4.50
	elevation	3	26.54	3	<0.001	20.54	1	2.55	1	0.110	0.55	3	15.69	3	0.001	9.69	3	108.34	3	<0.001	102.34
	ag.suitability	3	11.04	3	0.011	5.04	1	2.13	1	0.144	0.13	3	28.82	3	<0.001	22.81	3	11.56	3	0.009	5.56
	taxon		10.47	2	0.005	6.47		18.09	2	<0.001	14.09		4.05	2	0.131	0.06		44.59	2	<0.001	40.59
	latitudinal zone		5.37	1	0.020	3.37		3.83	1	0.501	1.83		6.19	1	0.013	4.19		61.77	1	<0.001	59.78
	LUPA		106.00	10	<0.001	86.00		103.12	10	<0.001	83.12		87.74	10	<0.001	67.44		89.84	10	<0.001	69.84
	LUPA:taxon		77.04	10	<0.001	57.04		105.94	10	<0.001	81.94		95.02	10	<0.001	75.02		74.99	10	<0.001	54.99
All-sites		statistics for main terms are the same as rows 13 – 19 of this table																			
	LUPA		94.73	6	<0.001	82.74		40.70	6	<0.001	28.70		0.37	6	0.588	-3.63		107.65	6	<0.001	95.65
	LUPA: latitudinal zone		66.16	5	<0.001	56.16		32.15	5	<0.001	22.15		4.29	5	0.508	-5.71		91.27	5	<0.001	81.27
All-sites	LUUI		329.08	8	<0.001	313.08		148.63	8	<0.001	132.63		78.07	8	<0.001	65.07		338.95	8	<0.001	322.95
	protection		2.31	1	0.128	0.31		2.38	1	0.123	0.38		0.40	1	0.529	-1.60		0.15	1	0.701	-1.85
	slope	1	0.56	1	0.455	-1.44	1	0.069	1	0.794	-1.93	1	0.91	1	0.339	-1.09	3	12.68	3	0.005	6.68
	elevation	3	28.11	3	<0.001	22.11	1	2.41	1	0.120	0.41	3	14.80	3	0.002	8.80	3	84.50	3	<0.001	78.50
	ag.suitability	3	11.29	3	0.010	5.29	1	2.02	1	0.155	0.02	3	30.43	3	<0.001	24.43	3	7.93	3	0.048	1.93
	taxon		10.69	2	0.005	6.70		24.46	2	<0.001	20.46		4.71	2	0.095	0.71		45.25	2	<0.001	41.25
	latitudinal zone		4.83	1	0.028	2.83		5.97	1	0.015	3.97		5.96	1	0.015	3.96		60.01	1	<0.001	58.01
	LUUI: protection		27.07	8	<0.001	12.07		39.75	8	<0.001	23.75		6.23	8	0.621	-9.77		52.51	8	<0.001	36.51

Supplementary Table 3 | Statistics from mixed effects models testing for effect of protection, using only data from sites matched by land use (as in main text Fig. 1d). Right hand column and χ^2,df and p-values given for removal of term specified. Protection was a two level factor specifying whether each site is protected vs unprotected, management category group was a four-level factor specifying whether each site was: unprotected, in a protected area with IUCN category I & II, IUCN category unknown or IUCN category III to VI. AGSZ was a five level factor for age and size groups (unprotected, small and young, small and old, large and young, large and old). Taxon was a three level factor for taxonomic groups (plants, vertebrates and invertebrates) and latitudinal zone was a two level factor (tropical and temperate). Agricultural suitability, slope and elevation were all continuous variables (for which cubic, quadratic and linear terms were tested sequentially) and the polynomial term retained in the final model is given in "poly". dAIC gives the change in AIC from a model with the term specified to one without.

		Species richness					Abundance					Rarefied richness					Endemicity				
**Dataset**	**Term removed**	**poly**	**χ^2**	**df**	**p**	**dAIC**	**poly**	**χ^2**	**df**	**p**	**dAIC**	**poly**	**χ^2**	**df**	**p**	**dAIC**	**poly**	**χ^2**	**df**	**p**	**dAIC**
Matched-sites	protection		1.28	1	0.258	-0.72		0.42	1	0.517	-1.58		0.31	1	0.589	-1.69		0.31	1	0.575	-1.69
	slope	1	0.13	1	0.714	-1.87	1	0.87	1	0.352	-1.13	2	4.01	1	0.045	2.01	3	10.74	1	0.001	8.74
	elevation	1	44.74	1	<0.001	42.74	1	4.57	1	0.033	2.57	3	5.65	1	0.017	3.65	2	46.33	1	<0.001	44.33
	ag.suitability	3	13.28	1	<0.001	11.28	1	3.98	1	0.046	1.97	3	8.69	1	0.003	6.69	3	9.70	1	1.839	7.70
Matched-sites	management category		3.24	3	0.356	-2.76		0.18	3	0.980	-5.81		1.88	3	0.598	-4.12		4.63	3	0.201	-1.37
	slope	1	0.21	1	0.649	-0.76	1	3.80	1	0.051	1.80	2	4.58	1	0.599	2.57	1	5.41	1	0.020	3.41
	elevation	1	41.07	1	<0.001	39.07	1	1.09	1	0.296	-0.91	3	3.86	1	0.049	1.86	1	93.88	1	<0.001	91.88
	ag.suitability	1	1.24	1	0.265	-0.76	1	3.92	1	0.048	1.92	1	8.79	1	0.003	6.78	1	0.22	1	0.637	-1.78
Matched-sites tropical	protection		0.04	1	0.846	-1.96		<0.01	1	0.999	-2.00		0.04	1	0.839	-1.96		0.20	1	0.658	-1.80
	slope	1	0.88	1	0.348	-1.12	3	7.84	1	0.005	5.84	2	15.92	1	<0.001	13.92	1	2.05	1	0.152	0.05
	elevation	3	13.89	1	<0.001	11.89	3	9.15	1	0.002	7.15	1	4.57	1	0.033	2.57	3	8.04	1	0.005	6.04
	ag.suitability	3	19.17	1	<0.001	17.17	3	4.22	1	0.040	2.22	3	3.89	1	0.049	1.89	2	6.58	1	0.010	4.58
Matched-sites temperate	protection		1.57	1	0.211	-0.43		1.08	1	0.299	-0.92		0.57	1	0.450	-1.43		2.23	1	0.136	0.23
	slope	1	0.07	1	0.799	-1.93	1	0.36	1	0.549	-1.64	1	1.61	1	0.205	-0.39	3	8.90	1	0.003	6.90
	elevation	1	19.25	1	<0.001	17.25	1	<0.01	1	0.926	-1.99	1	0.03	1	0.871	-1.97	3	7.89	1	0.005	5.89
	ag.suitability	1	0.29	1	0.593	-1.71	1	0.48	1	0.490	-1.52	1	0.82	1	0.365	-1.18	3	17.97	1	<0.001	15.97
Matched-sites plants	protection		0.55	1	0.457	-1.45		0.70	1	0.404	-1.30		4.48	1	0.034	2.48		0.60	1	0.440	-1.40
	slope	1	1.43	1	0.231	-0.57	3	7.50	1	0.006	5.50	1	0.35	1	0.553	-1.65	1	2.13	1	0.144	0.13
	elevation	2	9.38	1	0.002	7.38	1	5.45	1	0.020	3.45	1	2.94	1	0.087	0.94	3	7.76	1	0.005	5.76
	ag.suitability	1	5.13	1	0.023	3.13	1	0.70	1	0.401	-1.30	3	1.12	1	<0.001	9.08	1	2.76	1	0.097	0.76
Matched-sites invertebrates	protection		1.58	1	0.209	-0.42		0.95	1	0.329	-1.05		0.17	1	0.678	-1.83		1.10	1	0.295	-0.90
	slope	1	0.86	1	0.354	-1.14	1	0.36	1	0.551	-1.64	3	4.12	1	0.012	2.12	1	0.67	1	0.414	-1.33
	elevation	1	6.57	1	0.010	4.57	1	2.21	1	0.137	0.21	1	0.51	1	0.476	-1.49	1	0.40	1	0.528	-1.60
	ag.suitability	1	2.55	1	0.110	0.55	1	1.26	1	0.262	-0.74	1	1.15	1	0.284	-0.85	1	0.21	1	0.644	-1.79
Matched-sites vertebrates	protection		<0.01	1	0.996	-2.00		1.50	1	0.220	-0.50		1.99	1	0.159	-0.01		0.43	1	0.511	-1.57
	slope	1	1.41	1	0.235	-0.59	2	12.47	1	<0.001	10.47	2	4.63	1	0.031	2.63	3	11.03	1	<0.001	9.03
	elevation	3	4.18	1	0.041	2.18	1	0.99	1	0.319	-1.00	1	0.09	1	0.761	-1.91	3	19.81	1	<0.001	17.81
	ag.suitability	3	12.36	1	<0.001	10.36	3	6.71	1	0.010	4.71	1	0.42	1	0.519	-1.58	3	4.61	1	0.032	2.61
Matched-sites	AGSZ		16.22	4	0.003	8.22		3.70	4	0.448	-4.30		7.33	4	0.120	-0.67		7.73	4	0.102	-0.27
	taxon		8.29	2	0.016	4.29		13.46	2	0.001	9.46		2.91	2	0.233	-1.09		49.81	2	<0.001	45.81
	latitudinal zone		4.72	1	0.030	2.72		3.05	1	0.081	1.05		5.56	1	0.018	3.56		62.70	1	<0.001	60.70
	slope	1	0.04	1	0.841	-1.96	1	3.67	1	0.056	1.67	1	<0.01	1	0.952	-2.00	3	9.53	1	0.002	7.53
	elevation	2	7.29	1	0.007	5.29	1	0.91	1	0.339	-1.09	3	9.29	1	0.002	7.29	2	26.61	1	<0.001	24.61
	ag.suitability	3	15.58	1	<0.001	13.59	1	4.00	1	0.046	2.00	3	8.87	1	0.003	6.87	3	4.60	1	0.030	2.70
	AGSZ :taxon		10.04	8	0.262	-5.95		25.38	8	0.001	9.37		8.58	8	0.380	-7.42		16.64	8	0.034	0.65
	AGSZ: latitudinal zone		5.81	4	0.214	-2.19		7.19	4	0.126	-0.81		4.50	4	0.343	-3.50		3.28	4	0.511	-4.72

Supplementary Notes

List of data sources.

Adum GB, Eichhorn MP, Oduro W, Ofori-Boateng C, Rodel MO (2013) Two-stage recovery of amphibian assemblages following selective logging of tropical forests. *Conservation Biology* 27(2):354–363.

Ancrenaz M, Goossens B, Gimenez O, Sawang A, Lackman-Ancrenaz I (2004) Determination of ape distribution and population size using ground and aerial surveys: a case study with orang-utans in lower Kinabatangan, Sabah, Malaysia. *Animal Conservation* 7:375–385.

Arroyo J, Iturrondobeitia JC, Rad C, Gonzalez-Carcedo S (2005) Oribatid mite (Acari) community structure in steppic habitats of Burgos Province, central northern Spain. *Journal of Natural History* 39(39):3453–3470.

Azhar B, *et al.* (2013) The influence of agricultural system, stand structural complexity and landscape context on foraging birds in oil palm landscapes. *Ibis* 155(2):297–312.

Baeten L, Hermy M, Van Daele S, Verheyen K (2010) Unexpected understorey community development after 30 years in ancient and post-agricultural forests. *Journal of Ecology* 98(6):1447–1453.

Báldi A, Batáry P, Erdős S (2005) Effects of grazing intensity on bird assemblages and populations of Hungarian grasslands. *Agriculture Ecosystems & Environment* 108(3):251–263.

Banks JE, Sandvik P, Keesecker L (2007) Beetle (Coleoptera) and spider (Araneae) diversity in a mosaic of farmland, edge, and tropical forest habitats in western Costa Rica. *Pan-Pacific Entomologist* 83(2):152–160.

Berg A, Ahrne K, Ockinger E, Svensson R, Soderstrom B (2011) Butterfly distribution and abundance is affected by variation in the Swedish forest-farmland landscape. *Biological Conservation* 144(12):2819–2831.

Berry NJ, *et al.* (2010) The high value of logged tropical forests: lessons from northern Borneo. *Biodiversity and Conservation* 19(4):985–997.

Bicknell J, Peres CA (2010) Vertebrate population responses to reduced-impact logging in a neotropical forest. *Forest Ecology and Management* 259(12):2267–2275.

Billeter R, *et al.* (2008) Indicators for biodiversity in agricultural landscapes: a pan-European study. *Journal of Applied Ecology* 45(1):141–150.

Borges PAV, *et al.* (2006) Invasibility and species richness of island endemic arthropods: a general model of endemic vs. exotic species. *Journal of Biogeography* 33(1):169–187.

Bos MM, Steffan-Dewenter I, Tscharntke T (2007) The contribution of cacao agroforests to the conservation of lower canopy ant and beetle diversity in Indonesia. *Biodiversity and Conservation* 16(8):2429–2444.

Bouyer J, *et al.* (2007) Identification of ecological indicators for monitoring ecosystem health in the trans-boundary W Regional park: A pilot study. *Biological Conservation* 138(1-2):73–88.

Bragagnolo C, Nogueira AA, Pinto-da-Rocha R, Pardini R (2007) Harvestmen in an Atlantic forest fragmented landscape: Evaluating assemblage response to habitat quality and quantity. *Biological Conservation* 139(3-4):389–400.

Brearley FQ (2011) Below-ground secondary succession in tropical forests of Borneo. *Journal of Tropical Ecology* 27:413–420.

Castro-Luna AA, Sosa VJ, Castillo-Campos G (2007) Bat diversity and abundance associated with the degree of secondary succession in a tropical forest mosaic in south-eastern Mexico. *Animal Conservation* 10(2):219–228.

Chapman K, Reich P (2007) Land use and habitat gradients determine bird community diversity and abundance in suburban, rural and reserve landscapes of Minnesota, USA. *Biological Conservation* 135(4):527–541.

Chauvat M, Wolters V, Dauber J (2007) Response of collembolan communities to land-use change and grassland succession. *Ecography* 30(2):183–192.

Cleary DFR, *et al.* (2004) Diversity and community composition of butterflies and odonates in an ENSO-induced fire affected habitat mosaic: a case study from East Kalimantan, Indonesia. *Oikos* 105(2):426–446.

Cleary DFR, Mooers AO (2006) Burning and logging differentially affect endemic vs. widely distributed butterfly species in Borneo. *Diversity and Distributions* 12(4):409–416.

Davis ALV, Philips TK (2005) Effect of deforestation on a southwest Ghana dung beetle assemblage (Coleoptera: Scarabaeidae) at the periphery of Ankasa conservation area. *Environmental Entomology* 34(5):1081–1088.

Diekötter T, Walther-Hellwig K, Conradi M, Suter M, Frankl R (2006) Effects of landscape elements on the distribution of the rare bumblebee species Bombus muscorum in an agricultural landscape. *Biodiversity and Conservation* 15(1):57–68.

Dolia J, Devy MS, Aravind NA, Kumar A (2008) Adult butterfly communities in coffee plantations around a protected area in the Western Ghats, India. *Animal Conservation* 11(1):26–34.

Dures SG, Cumming GS (2010) The confounding influence of homogenising invasive species in a globally endangered and largely urban biome: Does habitat quality dominate avian biodiversity? *Biological Conservation* 143(3):768–777.

Faruk A, Belabut D, Ahmad N, Knell RJ, Garner TWJ (2013) Effects of Oil-Palm Plantations on Diversity of Tropical Anurans. *Conservation Biology* 27(3):615–624.

Farwig N, Sajita N, Boehning-Gaese K (2008) Conservation value of forest plantations for bird communities in western Kenya. *Forest Ecology and Management* 255(11):3885–3892.

Fayle TM, *et al.* (2010) Oil palm expansion into rain forest greatly reduces ant biodiversity in canopy, epiphytes and leaf-litter. *Basic and Applied Ecology* 11(4):337–345.

Felton AM, Engstrom LM, Felton A, Knott CD (2003) Orangutan population density, forest structure and fruit availability in hand-logged and unlogged peat swamp forests in West Kalimantan, Indonesia. *Biological Conservation* 114(1):91–101.

Fensham R, Dwyer J, Eyre T, Fairfax R, Wang J (2012) The effect of clearing on plant composition in mulga (Acacia aneura) dry forest, Australia. *Austral Ecology* 37(2):183–192.

Fiera C (2008) Preliminary data on the species diversity of Collembola (Hexapoda: Collembola) along an urban gradient in București. *Travaux du Museum National d'Histoire Naturelle "Grigore Antipa"* 51:363–367.

Franzén M, Nilsson SG (2008) How can we preserve and restore species richness of pollinating insects on agricultural land? *Ecography* 31(6):698–708.

García KP, Ortiz Zapata JC, Aguayo M, Elia G D' (2013) Assessing rodent community responses in disturbed environments of the Chilean Patagonia. *Mammalia* 77(2):195–204.

Gaublomme E, Hendrickx F, Dhuyvetter H, Desender K (2008) The effects of forest patch size and matrix type on changes in carabid beetle assemblages in an urbanized landscape. *Biological Conservation* 141(10):2585–2596.

Goulson D, *et al.* (2010) Effects of land use at a landscape scale on bumblebee nest density and survival. *Journal of Applied Ecology* 47(6):1207–1215.

Goulson D, Lye GC, Darvill B (2008) Diet breadth, coexistence and rarity in bumblebees. *Biodiversity and Conservation* 17(13):3269–3288.

Gray CL, Slade EM, Mann DJ, Lewis OT (2014) Do riparian reserves support dung beetle biodiversity and ecosystem services in oil palm-dominated tropical landscapes? *Ecology and Evolution* 4(7):1049–1060.

Hanley ME (2005) Unpublished data of bee diversity in UK croplands.

Hanley ME (2011) Unpublished data of bee diversity in UK croplands and urban habitats.

Hassan SN, *et al.* (2013) Human-induced Disturbances Influence on Bird Communities of Coastal Forests in Eastern Tanzania. *British Journal of Applied Science & Technology* 3(1):48–64.

Herrmann F, Westphal C, Moritz RFA, Steffan-Dewenter I (2007) Genetic diversity and mass resources promote colony size and forager densities of a social bee (Bombus pascuorum) in agricultural landscapes. *Molecular Ecology* 16(6):1167–1178.

Hietz P (2005) Conservation of vascular epiphyte diversity in Mexican coffee plantations. *Conservation Biology* 19(2):391–399.

Higuera D, Wolf JHD (2010) Vascular epiphytes in dry oak forests show resilience to anthropogenic disturbance, Cordillera Oriental, Colombia. *Caldasia* 32(1):161–174.

Hilje B, Aide TM (2012) Recovery of amphibian species richness and composition in a chronosequence of secondary forests, northeastern Costa Rica. *Biological Conservation* 146(1):170–176.

Hylander K, Nemomissa S (2009) Complementary Roles of Home Gardens and Exotic Tree Plantations as Alternative Habitats for Plants of the Ethiopian Montane Rainforest. *Conservation Biology* 23(2):400–409.

Hylander K, Nilsson C, Gothner T (2004) Effects of buffer-strip retention and clearcutting on land snails in boreal riparian forests. *Conservation Biology* 18(4):1052–1062.

Hylander K, Weibull H (2012) Do time-lagged extinctions and colonizations change the interpretation of buffer strip effectiveness? - a study of riparian bryophytes in the first decade after logging. *Journal of Applied Ecology* 49(6):1316–1324.

Ims RA, Henden JA (2012) Collapse of an arctic bird community resulting from ungulate-induced loss of erect shrubs. *Biological Conservation* 149(1):2–5.

Jonsell M (2012) Old park trees as habitat for saproxylic beetle species. *Biodiversity and Conservation* 21(3):619–642.

Kappes H, Katzschner L, Nowak C (2012) Urban summer heat load: meteorological data as a proxy for metropolitan biodiversity. *Meteorologische Zeitschrift* 21(5):525–528.

Kati V, Zografou K, Tzirkalli E, Chitos T, Willemse L (2012) Butterfly and grasshopper diversity patterns in humid Mediterranean grasslands: the roles of disturbance and environmental factors. *Journal of Insect Conservation* 16(6):807–818.

Knight ME, *et al.* (2009) Bumblebee nest density and the scale of available forage in arable landscapes. *Insect Conservation and Diversity* 2(2):116–124.

Kolb A, Diekmann M (2004) Effects of environment, habitat configuration and forest continuity on the distribution of forest plant species. *Journal of Vegetation Science* 15(2):199–208.

Kőrösi Á, Batáry P, Orosz A, Rédei D, Báldi A (2012) Effects of grazing, vegetation structure and landscape complexity on grassland leafhoppers (Hemiptera: Auchenorrhyncha) and true bugs (Hemiptera: Heteroptera) in Hungary. *Insect Conservation and Diversity* 5(1):57–66.

Kurz DJ, Nowakowski AJ, Tingley MW, Donnelly MA, Wilcove DS (2014) Forest-land use complementarity modifies community structure of a tropical herpetofauna. *Biological Conservation* 170:246–255.

Lantschner MV, Rusch V, Hayes JP (2012) Habitat use by carnivores at different spatial scales in a plantation forest landscape in Patagonia, Argentina. *Forest Ecology and Management* 269:271–278.

Lantschner MV, Rusch V, Peyrou C (2008) Bird assemblages in pine plantations replacing native ecosystems in NW Patagonia. *Biodiversity and Conservation* 17(5):969–989.

Letcher SG, Chazdon RL (2009) Rapid Recovery of Biomass, Species Richness, and Species Composition in a Forest Chronosequence in Northeastern Costa Rica. *Biotropica* 41(5):608–617.

Maeto K, Sato S (2004) Impacts of forestry on ant species richness and composition in warm-temperate forests of Japan. *Forest Ecology and Management* 187(2-3):213–223.

Malonza PK, Veith M (2012) *Amphibian community along elevational and habitat disturbance gradients in the Taita Hills, Kenya* Available at: http://proyectos.saber.ula.ve/index.php/herpetotropicos/article/view/4015.

Marshall EJP, West TM, Kleijn D (2006) Impacts of an agri-environment field margin prescription on the flora and fauna of arable farmland in different landscapes. *Agriculture Ecosystems & Environment* 113(1-4):36–44.

Mayfield MM, Ackerly D, Daily GC (2006) The diversity and conservation of plant reproductive and dispersal functional traits in human-dominated tropical landscapes. *Journal of Ecology* 94(3):522–536.

McCarthy JL, McCarthy KP, Fuller TK, McCarthy TM (2010) Assessing Variation in Wildlife Biodiversity in the Tien Shan Mountains of Kyrgyzstan Using Ancillary Camera-trap Photos. *Mountain Research and Development* 30(3):295–301.

McFrederick QS, LeBuhn G (2006) Are urban parks refuges for bumble bees Bombus spp. (Hymenoptera: Apidae)? *Biological Conservation* 129(3):372–382.

Meijer SS, Whittaker RJ, Borges PAV (2011) The effects of land-use change on arthropod richness and abundance on Santa Maria Island (Azores): unmanaged plantations favour endemic beetles. *Journal of Insect Conservation* 15(4):505–522.

Milder JC, *et al.* (2010) Effects of farm and landscape management on bird and butterfly conservation in western Honduras. *Ecosphere* 1(1):art2.

Muchane MN, *et al.* (2012) Land use practices and their implications on soil macro-fauna in Maasai Mara ecosystem. *International Journal of Biodiversity and Conservation* 4(13):500–514.

Munyekenye F, Mwangi E, Gichuki N (2008) Bird species richness and abundance in different forest types at Kakamega Forest, western Kenya. *Ostrich* 79(1):37–42.

Naidoo R (2004) Species richness and community composition of songbirds in a tropical forest-agricultural landscape. *Animal Conservation* 7:93–105.

Nakamura A, Proctor H, Catterall CP (2003) Using soil and litter arthropods to assess the state of rainforest restoration. *Ecological Management & Restoration* 4(Supplement):S20–S28.

Naoe S, Sakai S, Masaki T (2012) Effect of forest shape on habitat selection of birds in a plantation-dominant landscape across seasons: comparison between continuous and strip forests. *Journal of Forest Research* 17(2):219–223.

Neuschulz EL, Botzat A, Farwig N (2011) Effects of forest modification on bird community composition and seed removal in a heterogeneous landscape in South Africa. *Oikos* 120(9):1371–1379.

Nicolas V, Barriere P, Tapiero A, Colyn M (2009) Shrew species diversity and abundance in Ziama Biosphere Reserve, Guinea: comparison among primary forest, degraded forest and restoration plots. *Biodiversity and Conservation* 18(8):2043–2061.

Norfolk O, Eichhorn MP, Gilbert F (2013) Traditional agricultural gardens conserve wild plants and functional richness in arid South Sinai. *Basic and Applied Ecology* 14(8):659–669.

Numa C, Verdu JR, Rueda C, Galante E (2012) Comparing Dung Beetle Species Assemblages Between Protected Areas and Adjacent Pasturelands in a Mediterranean Savanna Landscape. *Rangeland Ecology & Management* 65(2):137–143.

O'Connor TG (2005) Influence of land use on plant community composition and diversity in Highland Sourveld grassland in the southern Drakensberg, South Africa. *Journal of Applied Ecology* 42(5):975–988.

O'Dea N, Whittaker RJ (2007) How resilient are Andean montane forest bird communities to habitat degradation? *Biodivers Conserv* 16(4):1131–1159.

Oke OC, Chokor JU (2009) The effect of land use on snail species richness and diversity in the tropical rainforest of south-western Nigeria. *African Scientist* 10(2):95–108.

Osgathorpe LM, Park K, Goulson D (2012) The use of off-farm habitats by foraging bumblebees in agricultural landscapes: implications for conservation management. *Apidologie* 43(2):113–127.

Otto CRV, Roloff GJ (2012) Songbird response to green-tree retention prescriptions in clearcut forests. *Forest Ecology and Management* 284:241–250.

Parra-H A, Nates-Parra G (2007) Variation of the orchid bees community (Hymenoptera : Apidae) in three altered habitats of the Colombian "llano" piedmont. *Revista de Biología Tropical* 55(3-4):931–941.

Phalan B, Onial M, Balmford A, Green R (2011) Reconciling Food Production and Biodiversity Conservation: Land Sharing and Land Sparing Compared. *Science* 333(6047):1289–1291.

Politi N, Hunter M Jr, Rivera L (2012) Assessing the effects of selective logging on birds in Neotropical piedmont and cloud montane forests. *Biodiversity and Conservation* 21(12):3131–3155.

Pons P, Wendenburg C (2005) The impact of fire and forest conversion into savanna on the bird communities of West Madagascan dry forests. *Animal Conservation* 8:183–193.

Poveda K, Martinez E, Kersch-Becker M, Bonilla M, Tscharntke T (2012) Landscape simplification and altitude affect biodiversity, herbivory and Andean potato yield. *Journal of Applied Ecology* 49(2):513–522.

Power EF, Kelly DL, Stout JC (2012) Organic Farming and Landscape Structure: Effects on Insect-Pollinated Plant Diversity in Intensively Managed Grasslands. *PLoS ONE* 7(5) e38073.

Power EF, Stout JC (2011) Organic dairy farming: impacts on insect-flower interaction networks and pollination. *Journal of Applied Ecology* 48(3):561–569.

Proenca VM, Pereira HM, Guilherme J, Vicente L (2010) Plant and bird diversity in natural forests and in native and exotic plantations in NW Portugal. *Acta Oecologica-International Journal of Ecology* 36(2):219–226.

Redpath N, Osgathorpe LM, Park K, Goulson D (2010) Crofting and bumblebee conservation: The impact of land management practices on bumblebee populations in northwest Scotland. *Biological Conservation* 143(2):492–500.

Römbke J, Schmidt P, Höfer H (2009) The earthworm fauna of regenerating forests and anthropogenic habitats in the coastal region of Paraná. *Pesquisa Agropecuaria Brasileira* 44(8):1040–1049.

Safian S, Csontos G, Winkler D (2011) Butterfly community recovery in degraded rainforest habitats in the Upper Guinean Forest Zone (Kakum forest, Ghana). *Journal of Insect Conservation* 15(1-2):351–359.

Sakchoowong W, Nomura S, Ogata K, Chanpaisaeng J (2008) Diversity of pselaphine beetles (Coleoptera: Staphylinidae: Pselaphinae) in eastern Thailand. *Entomological Science* 11(3):301–313.

Santana J, Porto M, Gordinho L, Reino L, Beja P (2012) Long-term responses of Mediterranean birds to forest fuel management. *Journal of Applied Ecology* 49(3):632–643.

Savage J, Wheeler TA, Moores AMA, Taillefer AG (2011) Effects of Habitat Size, Vegetation Cover, and Surrounding Land Use on Diptera Diversity in Temperate Nearctic Bogs. *Wetlands* 31(1):125–134.

Sheil D, *et al.* (2002) *Exploring biological diversity, environment and local people's perspectives in forest landscapes: Methods for a multidisciplinary landscape assessment* (Center for International Forestry Research (CIFOR), Jakarta).

Soh MCK, Sodhi NS, Lim SLH (2006) High sensitivity of montane bird communities to habitat disturbance in Peninsular Malaysia. *Biological Conservation* 129(2):149–166.

Stouffer PC, Johnson EI, Bierregaard RO Jr, Lovejoy TE (2011) Understory Bird Communities in Amazonian Rainforest Fragments: Species Turnover through 25 Years Post-Isolation in Recovering Landscapes. *PLoS ONE* 6(6):e20543.

Ström L, Hylander K, Dynesius M (2009) Different long-term and short-term responses of land snails to clear-cutting of boreal stream-side forests. *Biological Conservation* 142(8):1580–1587.

Struebig MJ, Kingston T, Zubaid A, Mohd-Adnan A, Rossiter SJ (2008) Conservation value of forest fragments to Palaeotropical bats. *Biological Conservation* 141(8):2112–2126.

Suarez-Rubio M, Thomlinson JR (2009) Landscape and patch-level factors influence bird communities in an urbanized tropical island. *Biological Conservation* 142(7):1311–1321.

Summerville KS, Conoan CJ, Steichen RM (2006) Species traits as predictors of lepidopteran composition in restored and remnant tallgrass prairies. *Ecological Applications* 16(3):891–900.

Summerville KS, Crist TO (2002) Effects of timber harvest on forest Lepidoptera: community, guild and species responses. *Ecological Applications* 12(3):820–835.

Threlfall CG, Law B, Banks PB (2012) Sensitivity of insectivorous bats to urbanization: Implications for suburban conservation planning. *Biological Conservation* 146(1):41–52.

Tonietto R, Fant J, Ascher J, Ellis K, Larkin D (2011) A comparison of bee communities of Chicago green roofs, parks and prairies. *Landscape and Urban Planning* 103(1):102–108.

Turner EC, Foster WA (2009) The impact of forest conversion to oil palm on arthropod abundance and biomass in Sabah, Malaysia. *Journal of Tropical Ecology* 25:23–30.

Unpublished data of reptilian and amphibian diversity in six countries in Central America (2010).

Vallan D (2002) Effects of anthropogenic environmental changes on amphibian diversity in the rain forests of eastern Madagascar. *Journal of Tropical Ecology* 18:725–742.

Vanbergen AJ, Woodcock BA, Watt AD, Niemela J (2005) Effect of land-use heterogeneity on carabid communities at the landscape scale. *Ecography* 28(1):3–16.

Verdasca MJ, *et al.* (2012) Forest fuel management as a conservation tool for early successional species under agricultural abandonment: The case of Mediterranean butterflies. *Biological Conservation* 146(1):14–23.

Walker S, Wilson DJ, Norbury G, Monks A, Tanentzap AJ (2014) Complementarity of indigenous flora in shrublands and grasslands in a New Zealand dryland landscape. *New Zealand Journal of Ecology* 38(2):230–241.

Weller B, Ganzhorn JU (2004) Carabid beetle community composition, body size, and fluctuating asymmetry along an urban-rural gradient. *Basic and Applied Ecology* 5(2):193–201.

Williams CD, Sheahan J, Gormally MJ (2009) Hydrology and management of turloughs (temporary lakes) affect marsh fly (Sciomyzidae: Diptera) communities. *Insect Conservation and Diversity* 2(4):270–283.

Woinarski JCZ, *et al.* (2009) Fauna assemblages in regrowth vegetation in tropical open forests of the Northern Territory, Australia. *Wildlife Research* 36(8):675–690.

Yan XL, Bao WK, Pang XY, Zhang NX, Chen JQ (2013) Regeneration strategies influence ground bryophyte composition and diversity after forest clearcutting. *Annals of Forest Science* 70(8):845–861.

Zaitsev AS, Wolters V, Waldhardt R, Dauber J (2006) Long-term succession of oribatid mites after conversion of croplands to grasslands. *Applied Soil Ecology* 34(2-3):230–239.

Supplementary Methods

Data. The PREDICTS (Projecting Responses of Ecological Diversity in Changing Terrestrial Systems) database collates spatial comparisons of community composition and site-level biodiversity from terrestrial sites around the world[2]. The primary aim of the PREDICTS project is to model and project how biodiversity in terrestrial communities responds to human activity[2,3]. We used data entered into the PREDICTS database up to the 13th November 2014. These data were obtained from studies identified through a review of conservation and ecology literature (including primary literature and databases) and requests to authors[2]. Criteria for inclusion in the dataset were 1) data were published, in press or obtained using a published methodology 2) sampling took place post 2000, 3) studies sampled more than one land use, and 4) geographical coordinates of sampling locations are available[2]. Data on coordinates and site specific abundances or presence/absence data for individual species were obtained from the original publication or directly from the authors.

For sites where data on the abundance of individuals in each species were available we calculated the species richness, total abundance, rarefaction-based richness (hereafter 'rarefied richness') and mean community endemicity (1/community weighted mean range size (log10 km^2), hereafter endemicity). We selected these response variables as species richness and total abundance are easily understood by a broad audience, and because endemicity and rarefied richness indicate aspects of ecological communities that are often considered to be a conservation priority (higher numbers of species with small range sizes and a higher "concentration" of species for a given number of individuals). Species range sizes were measured as in ref 4, using the total land area of the 1° grid cells with records in the Global Biodiversity Information Facility (GBIF) database (http://www.gbif.org/, downloaded August 2014). Where range size data was not available for one or more of the species' sampled, the community mean range size was calculated from species for which data where available. However, to check that these missing data did not affect our results, we also repeated our analyses using only the sites for which at least 80% of the individuals had known range data (see sensitivity analyses below). We recognise that the numbers of species records in GBIF are not consistent across geographic areas and taxonomic groups. However, most studies whose data we included in our analysis sampled a relatively confined taxonomic group and geographic spread. As our analyses used mixed-effects models (see below), our results are driven by within-study differences between protected and unprotected sites, which are much less likely to be affected by the biases in the GBIF database. In addition, we have

also found that differences in the grain size used (1, 0.1 or 0.01° grid cells) do not affect the estimates of species' range size, and that our GBIF occupancy measure gives similar range estimates to those derived from IUCN extent-of-occurrence maps[5].

To calculate rarefied richness, we applied the same rarefaction procedure to each study that had data on species' abundance. First, we calculated the minimum abundance, n_{min}, recorded at any one site in the study. We then took a random sample (without replacement) of n_{min} individuals from the pool of individuals recorded at each site in that study. Rarefied richness was the count of species in this random sample. We repeated this process 1000 times and calculated one overall mean rarefied richness, rounded to the nearest integer. For some studies we only had data on species occurrence; a binary variable describing whether or not a species was detected in the sample at a given site, for all species observed in that study. For these sites we calculated only species richness and endemicity. Site land use was classified from habitat descriptions in the literature and from contact with authors, as primary vegetation, secondary vegetation (mature, intermediate, young), plantation, pasture, cropland or urban. We used these habitat descriptions to allocate land use rather than obtaining information on land cover from global satellite imagery products, as the latter cannot easily distinguish certain land uses (e.g. forest and plantation, grassland and pasture), and generally have lower spatial and temporal resolution. A repeatability analysis was carried out on a subset of the PREDICTS data to ensure that the land use classifications were consistently applied – further details are given in ref 2. In addition to land use, levels of intensity of use (minimal, light, intense, or unknown [excluded from these analyses]) were assigned based on the literature and contact with authors. Further details of the PREDICTS database and definitions of land use and use intensity are given in ref 2.

The PREDICTS database does not target protected area studies specifically. Only 10 of 115 sources included in our analyses mentioned that they were intentionally comparing sites within a protected area and those outside. The overlap between the sources used in our analysis and other meta-analyses of protected area effectiveness was very low (no overlap with the sources listed by Geldmann *et al.*[6] and only 6 sources shared with Coetzee *et al.*[7]), so our data provide a new body of evidence that has not previously been used to answer questions about the biodiversity within protected areas.

Information on protected areas was obtained from the July 2014 release of the World Database on Protected Areas (WDPA)[1]. We used all protected areas with "designated" or "proposed" status, excluded UNESCO Man and Biosphere reserves because these include buffer and transition areas which do not meet the IUCN definition of protected areas. For

protected areas with known locations and reported areas but unknown boundaries (14 of 359 protected areas, containing 245 of 6531 sites in our analyses [3.8%]), we created circular buffers with an area equal to the reported value. For each PREDICTS site falling within a protected area, we extracted the year the protected area was designated (STATUS_YR), size (GIS area) and IUCN Protected Area Management Category (henceforth IUCN category). Where a PREDICTS site fell into more than one protected area, we selected the size of the largest protected area, the status year of the oldest protected area and the IUCN category of the protected area with the most restrictive management objectives (where Ia > Ib > II > III > IV > V > VI). Where countries have not used the IUCN system to classify their protected areas, or the IUCN category was unreported (IUCN_CAT = "Not Applicable" or "Not Reported"), we set our IUCN category variable to "unknown". As studies were carried out in different years, we calculated the duration of protection at each site when it was sampled: the difference between the year of earliest sampling at the site and the STATUS_YR. If STATUS_YR was unknown, we treated this as missing data and set protected area age to "NA"; these data points were excluded from analyses of age and size class but included in all other analyses. Sites with a duration of protection less than or equal to zero were considered to be unprotected.

We assessed whether our sites and the protected areas sampled are randomly placed with respect to latitude and longitude and found no bias towards higher or lower latitudes ($P = 0.51$, $\chi^2 = 0.44$, df = 1) or longitudes ($P = 0.12$, $\chi^2 = 2.47$, df = 1), using a mixed effect model approach with either latitude or longitude as a response variable, protection status (in vs out) as a fixed effect and study as a random effect (with a study-specific gradient for protection).

We obtained data on three covariates that may affect biodiversity response: 1) Mean elevation at 30 arc-second (approximately 1 km^2 grid cell size) resolution[8]; 2) Slope, calculated from elevation data at 30 arc-second (approximately 1 km^2 grid cell size) resolution; 3) Agricultural suitability, available at 5 arc-minute (approximately 10 km^2 grid cell size) resolution[9]. For each site, we calculated the mean elevation and slope across a buffer with 1 km radius. Due to the lower spatial resolution of the dataset, for agricultural suitability we took the absolute value of the raster at each site location. There are other factors that may influence biodiversity that we purposely did not incorporate as covariates in our models, such as human population density, accessibility (travel time to the nearest city > 5000 people), distance to market, or distance to roads. These factors may have been influenced by the presence of a protected area, so we did not consider them to be fully

independent confounding effects. Other potential confounding variables, such as evapotranspiration, precipitation and mean temperature are included in the agricultural suitability layer[9] and thus including these variables separately would entail collinearity between explanatory and confounding variables. The latitudinal zone (tropical or temperate) of each site was allocated based on whether sites fell between tropical latitudes (approximately ± 23.5^{o} from the equator[10,11], Fig. 1). All studies fell into either the tropical or temperate zone.

All spatial calculations were carried out in ESRI ArcMap (version 10.0, ref 12) using an equal-area Mollweide projection. The package rgdal[13] was used to plot data in R.

All-sites data. To test whether the effect of protected areas differed between land uses, we included data from all PREDICTS studies with at least one site inside and one site outside a protected area (Fig. 1B). To ensure that there were sufficient sites to make a robust estimate of the effects of land use inside and outside protected areas we had to retain all sites within these studies. To avoid artefactual differences arising because of comparisons between sites with extremely large distances between them, any sites that were on average more than 150 km away from other sites in their study were dropped. This distance was selected as it formed a clear break in the histogram of distances between sites, above which frequencies were consistently low. Each site was allocated to a unique taxonomic group (vertebrates, invertebrates, or plants), with two studies that sampled more than one major taxonomic group at each site divided accordingly. The all-sites data included data for 6531 sites from 156 studies and covers 13,669 species, 48 countries, 101 ecoregions, 13 biomes[14], 23 hotspots and 359 protected areas.

Matched-sites data. In the all-sites data, sites within a protected area may be in a different land use to sites outside a protected area, but within the same study. We therefore applied an additional, stricter data selection process to assess whether protected areas have an effect within a given land use and which protected area characteristics alter this effect. The matched-sites data retained only sites within the same land uses that occurred both inside and outside the protected area(s) within any given study (Fig. 1d). The matched-sites data included data for 5015 sites from 144 studies and covers 12,898 species, 44 countries, 85 ecoregions, 21 hotspots and 313 protected areas.

Statistical analyses. Both abundance and occurrence records will have been influenced by study-specific sampling methodologies; we used mixed effect models to control for these differences. We compared nested random factor structures containing source ID, study ID (studies were separated by sampling method, and more than one study may have been presented within the same source), and block (derived from grouping structures where reported in the original study). See equations A to E (below) for model structures used in each analysis. We also compared random factor structures with and without a random slope for protection status (two-level factor, protected vs unprotected) or management category (four-level factor: unprotected, IUCN category I & II, IUCN category unknown, and IUCN category II to VI) at the study level, depending on which of these factors was included as a fixed effect. The best random factor structure was selected using Akaike Information Criterion (AIC) values.

The geographical coverage of the PREDICTS database did not allow us to apply the strict counterfactual matching approach, so instead we included slope, elevation and agricultural suitability as covariates in our models. There are other factors that may influence biodiversity that we purposely did not match for, or incorporate as covariates in our models, such as fragmentation, human population density or distance to roads. These may have been influenced by the presence of a protected area, so we did not consider them to be fully independent confounding effects.

Selection of fixed effects was based on p-values, using a threshold of 0.05. Changes in AIC for each term dropped from the model are also given in results tables for comparison. As a result of corrections for sampling efforts, not all abundance values were integers, so we log-transformed abundance data. Species richness and rarefied richness data were modelled using Poisson errors (log link function). Observation level random effects were included where needed to account for overdispersion. Any studies that focussed on only one species [7 studies (4% of all studies) accounting for 2% of all the sites in the all-sites data] were excluded from analyses where species richness or rarefied richness was the response variable. Sites without complete data were dropped.

All analyses were carried out in R[15] using the lme4[16] and influence.ME[17] packages.

Analyses for "Local biodiversity inside and outside protected areas" Models were run on the all-sites data and included protection status (PS, inside versus outside a protected area) as a fixed effect, with the confounding variables agricultural suitability (AG), elevation (EL) and slope (SL) included as covariates. Equation A gives an example of the full model structure for the response variable species richness (SR), where the random structure includes study (SS), block (SSB) and site (SSBS). Note that when random intercepts are strictly nested, the following two ways of specifying the random-effects structure are exactly equivalent: (1|SS) + (1|SSB) + (1|SSBS) and (1|SS/SSB/SSBS):

```
M1 <- glmer(SR ~ PS + poly(AG,3) + poly(SL,3) + poly(EL,3) +
        (1+PS|SS) + (1|SSB) + (1|SSBS), family = "poisson")        (A)
```

To test for differences between IUCN categories we replaced the variable describing protection status with one describing management category group (factor with four levels; unprotected, IUCN category I & II, IUCN category III to VI and IUCN category unknown), again including all three confounding variables as fixed effects. To confirm that results were not driven by differences in underlying datasets, the influence of small studies or studies specifically focussing on protected areas, we repeated these models with appropriate subsets of the data (see sensitivity analyses below).

Analyses for "Effects of protection within and among land uses" Biodiversity measures and protection status across different land-uses were also modelled using the all-sites data. Here we included PS, land use (LU_8: eight level factor; primary, secondary mature, secondary intermediate, secondary young, plantation, cropland, pasture and urban), taxonomic group (TG), latitudinal zone (LZ), AG, SL, EL, and the land-use: protection interaction as fixed effects in our model selection process. Equation B gives an example of this full model structure for SR, with the random structure as above:

```
M1 <- glmer(SR ~ LU_8 + PS + TG + LZ + LU_8:PS +
        poly(AG,3) + poly(SL,3) + poly(EL,3) +
        (1+PS|SS) + (1|SSB) + (1|SSBS), family = "poisson")        (B)
```

To achieve sufficient numbers of observations in each comparison group when modelling the effects of land use intensity, we created a nine-level factor (LUUI) expressing the land use

intensity at each site, and whether the land use was primary vegetation, secondary vegetation (any age class) or human-dominated (plantation, cropland, pasture, urban). We then tested for an interaction between this nine-level factor and PS. Equation C gives an example of this full model structure for SR, with the same random factor structure as in equation B above:

```
M1 <- glmer(SR ~ PS + LUUI + LUUI:PS + TG + LZ +
        poly(AG,3) + poly(SL,3) + poly(EL,3) +
        (1+PS|SS) + (1|SSB) + (1|SSBS), family = “poisson”)          (C)
```

To model responses to protection across land-uses in different LZ and TG we created a compound variable (LUPA) with six levels, one for each combination of land use (primary, secondary or human dominated) and PS. We then tested for the interaction between this variable and either TG (as in equation D) or LZ.

```
M1 <- glmer(SR ~ LUPA + TG + LZ + LUPA:TG
        poly(AG,3) + poly(SL,3) + poly(EL,3) +
        (1+PS|SS) + (1|SSB) + (1|SSBS), family = “poisson”)          (D)
```

To confirm that results were not driven by differences in underlying datasets, the influence of small studies or studies specifically focussing on protected areas, we repeated these models with appropriate subsets of the data (see sensitivity analyses below).

Analyses for “Effect of protection when sites are matched by land use” Using the matched-sites data, we re-ran the same models used for the first set of analyses (see equation A above), with either protection status or management category group as a fixed effect, and subsetting the data into latitudinal zones or taxonomic groups as required.

In addition, to further assess whether particular protected area characteristics might affect the biodiversity response when matching for land use, we ran models specifying the age-size class of the protected areas (AGSZ, a factor with four levels; young (< 20 years) and small (< 400 km^2), young and large (400 – 12,000 km^2), old (20 – 85 years) and young, old and large) and including land use (LU) as a crossed random factor.

Equation E gives an example of this full model structure for the response variable species richness (SR), where the random structure includes study (SS), block (SSB) and site (SSBS):

```
M1 <- glmer(SR ~ AGSZ + LZ + TG + AGSZ:LZ + AGSZ:TG +
        poly(AG,3) + poly(SL,3) + poly(EL,3) +
        (1 |SS) + (1|SSB) + (1|SSBS) + (1|LU), family = "poisson")
```
(E)

We further assessed the importance of land use versus protection in explaining differences in local biodiversity by comparing the R^2 values of a model with only land use or protection as fixed effects and the full model (land use, protection and their interaction). We used the marginal R^2 value following methods described by Nakagawa & Schielzeth[18]. 90% (species richness), 90% (abundance) and 86% (endemicity) of the variance explained by the full model (with land use, protection and their interaction) were provided by a model with just the land-use term.

Analyses for "Estimating the global effectiveness of protected areas" If protected areas are entirely effective, sites inside protected areas should have the species richness and abundance of sites in minimally-used primary vegetation regardless of land use. If protected areas are not at all effective, their species richness and abundance should average the same as sites in the same land use outside protected areas. These endpoints delimit a natural scale on which to estimate protected area effectiveness, *e*.

We assume that an estimate of the state of global terrestrial biodiversity, expressed as a proportion of that found in minimally-used primary vegetation, is made up of the biodiversity inside protected areas and outside protected areas. We take *i* as the mean value of a biodiversity measure of sites inside protected areas, expressed as a proportion of that found in minimally-used primary vegetation, and *o* as the corresponding mean of sites outside expressed in the same way. If *a* is the proportion of the Earth's terrestrial surface inside protected areas and *r* the global average reduction in the mean site-level of the biodiversity measure caused by human pressures, then the state of global terrestrial biodiversity can be expressed as:

$$1 - r = ai + (1 - a)*o \qquad \text{(F)}$$

Newbold *et al.*[3] estimated *r* to be 0.129 for species richness and 0.126 for abundance for the year 2005 (we use values not weighted by spatial variation in species richness, as we did not weight sites by spatial variation in species richness in our analyses). Juffe-Bignoli *et al.*[19]

report that *a* was around 0.154 in 2014. To obtain an estimate of biodiversity in protected relative to unprotected sites (i.e. estimates of *i*/*o*), we used the coefficients from the model described by equation B as follows:

$$i = \beta_{pri\_in}*p\_pri\_in + \beta_{sec\_in}*p\_sec\_in + \beta_{crop\_in}*p\_crop\_in + \beta_{pas\_in}*p\_pas\_in + \beta_{urb\_in}*p\_urb\_in \quad (G)$$

$$o = 1*p\_pri\_out + \beta_{sec\_out}*p\_sec\_out + \beta_{crop\_out}*p\_crop\_out + \beta_{pas\_out}*p\_pas\_out + \beta_{urb\_out}*p\_urb\_out \quad (H)$$

where β_{x_in} is the coefficient for land use *x* inside protected areas and p_*x*_in is the proportion of total land area inside protected areas that is land use *x*, and likewise for outside protected areas.

To calculate the proportion of total land area in each land use, we used a fine-grained (30 arc-seconds; approximately 1 km) resolution land use map derived by downscaling the Harmonised Land Use dataset[20]. The downscaling algorithm used the Harmonised Land Use dataset[21] (50 km resolution) and higher resolution (1 km) predictor covariates describing climate (evapotranspiration[22], temperature and precipitation[23], topographic wetness[24]), landform and substrate (slope[24], soil carbon[25]), anthropogenic factors (accessibility[26], population density[27]) and land cover[28]. Pixels under water[29] or permanent ice cover (Rock and Ice classification in[14]) were masked out of these data layers. Initially, a GAM with a quasibinomial error structure and logistic link was fitted to each of the five coarse grained Harmonised Land Use datasets (response variables) using the fine-grained covariates as predictors. The fine-grained fitted values from these GAMs were then rescaled multiplicatively so that the aggregated mean for each coarse-grained cell matched that of the Harmonised Land Use estimate. We then passed these rescaled fitted values to a constrained optimisation algorithm to produce an estimate of land use for each fine-grained grid cell that obeyed the constraints of being in the range of 0 - 1 and where all five estimates (one for each of five land use types: primary habitat, secondary habitat, cropland, pasture and urban) within a cell sum to 1. These estimates were then fed back into the GAMs as response variables and the procedure iterated until a convergence criterion was met. The result is fine-grained downscaled proportion of five land use types that sum to 1 within each grid cell. This analysis was performed on the entire 2005 global data layer of the Harmonised Land Use

dataset to give estimates of the proportion of all five land uses from the Harmonised Land Use dataset for every fine grained terrestrial grid cell between 90°N and 60°S.

As the downscaled proportions of land use do not separate different ages of secondary vegetation, we re-ran the models including land use (equation B) using the all-sites data, but having combined all ages of secondary vegetation to give land use as a 6 level factor (primary, secondary, plantation, pasture, cropland and urban).

We then solved equation F for *i* and *o* and calculated the effectiveness of protected areas, *e*, from:

$$e = 1 - (1 - i)/(1 - o) \quad \text{(I)}$$

We calculated 95% confidences intervals using the upper and lower bounds for the estimate of site level biodiversity in unprotected and protected sites relative to primary unprotected.

Analyses for "How to improve the global protected area network". We used the models of biodiversity response to different management category groups to obtain an estimate of effectiveness, *e,* under the hypothetical circumstance that all existing protected areas showed the same biodiversity response as those in IUCN category I & II ($e_{\text{I \& II}}$). We assumed that the observed differences in local biodiversity measures among management category groups could be obtained by altering management objectives. However, some protected areas (e.g. in IUCN category I & II) have been specifically located in areas of high species richness, which will contribute to observed differences. We used the same effectiveness calculations described above, but *i/o* was given by the estimates for biodiversity at unprotected sites and those in protected areas with IUCN category I & II (from GLMMs with IUCN category as a fixed effect, equation A). We also obtained an estimate of *e* given a value of *i/o* derived from the estimates for unprotected sites and sites in IUCN categories III to VI ($e_{\text{III to VI}}$). Our data were insufficiently resolved to test for effects of management category group in different land uses, so we could not adjust these estimates to reflect the distribution of land uses within PAs.

We then estimated the increase in protected area required to achieve the same biodiversity outcome as that given if all current protected areas are assigned IUCN category I & II. To do this, we used equation F to estimate the total biodiversity loss, *r*, if the area under protection, *a*, remains at 15.4%, the value of biodiversity outside protected areas relative to pristine, *i*, is the same as that estimated given the current protected area network and the biodiversity at unprotected relative to protected sites, *i/o*, is given by the difference between

unprotected sites and those in IUCN category I & II. We then rearranged equation F to estimate the proportion of land area (*a*) that would be needed given this new value of *r*, given the same value of biodiversity outside protected areas relative to pristine, *i*, as that estimated given the current protected area network and where *i/o* was determined by the difference between unprotected and protected sites given the current network of protected areas with a mix of IUCN categories.

Sensitivity Analyses. We carried out four sets of analyses to explore whether our results were sensitive to particular features of the data:

1) Were differences between species richness and rarefied richness caused by differences in the data used? We removed data from sites where total species richness was known, but not the abundance of individuals from each species; these are the sites for which rarefied richness cannot be calculated. When land use was not included in the models, sites inside protected areas still showed higher species richness than those outside (Supplementary Tables 1 and 2). The only differences were that the estimate for plant species richness had much larger confidence intervals, and that the species richness of vertebrates and invertebrates were no longer significantly higher at sites inside protected areas, though there was still a positive trend towards this. When land use was included within the model, the trends were broadly similar to those found with the full species richness data set; protection status made the largest difference to sites in human-dominated land uses.
2) Is a publication bias towards studies reporting a positive effect of protection affecting our results? 9% of our studies were designed to compare biodiversity inside and outside a protected area and could potentially bias results. To address this possible sampling bias, we re-ran our models without studies that explicitly focussed on protected area effects. Again, no notable changes in the results occurred (see Supplementary Tables 1 and 2).
3) Is a publication bias towards studies reporting a strong effect of land use affecting our results? Small studies are more likely to suffer from publication bias towards significant results[30] and therefore a strong effect of land use, so we re-ran the models used to produce Fig. 2 and 3 without small studies (those with fewer than 10 sites, 32 studies or 13% of all studies). Apart from small increases in the size of some confidence intervals, there were no notable changes in the results (see Supplementary Tables 1 and 2).

4) Does incomplete data on range size affect our results? Our measure of endemicity is based on range sizes derived from GBIF data and not available for all species. We re-ran the analyses of endemicity (Fig. 2 and 3), using only data from the sites for which at least 80% of the individuals had known ranges. Overall, results were very similar (Supplementary Tables 1 and 2), although confidence intervals were wider. We have also checked that ranges for vertebrates derived from GBIF and IUCN data are broadly comparable for species in the PREDICTS database.

Supplementary References

1. IUCN and UNEP. The World Database on Protected Areas (WDPA), July 2014. <www.protectedplanet.net> (UNEP-WCMC, 2014).
2. Hudson, L. N. *et al.* The PREDICTS database: a global database of how local terrestrial biodiversity responds to human impacts. *Ecol. Evol.* **4**, 4701–4735 (2014).
3. Newbold, T. *et al.* Global effects of land use on local terrestrial biodiversity. *Nature* **520**, 45–50 (2015).
4. Newbold, T. *et al.* A global model of the response of tropical and sub-tropical forest biodiversity to anthropogenic pressures. *Proc. R. Soc. B Biol. Sci.* **281**, 20141371 (2014).
5. Newbold, T. *et al.* Global patterns of terrestrial assemblage turnover within and among land uses. *Ecography* < Global patterns of terrestrial assemblage turnover within and among land uses DOI:10.1111/ecog.01932 (2016).
6. Geldmann, J. *et al.* Effectiveness of terrestrial protected areas in reducing habitat loss and population declines. *Biol. Conserv.* **161**, 230–238 (2013).
7. Coetzee, B. W. T., Gaston, K. J. & Chown, S. L. Local scale comparisons of biodiversity as a test for global protected area ecological performance: a meta-analysis. *PLoS ONE* **9**, e105824 (2014).
8. Danielson, J. J. & Gesch, G. B. *Global multi-resolution terrain elevation data 2010 (GMTED2010).* US Geol. Surv. Open-File Rep. 2011–1073, 26 p (2011).
9. Fischer, G., van Velthuizen, H. T., Shah, M. M. & Nachtergaele, F. O. *Global Agro-Ecological Assessment for Agriculture in the 21st Century: Methodology and Results. Plate 46 (Suitability for rain-fed crops (maximizing technology mix)).* (International Institute for Applied Systems Analysis and Food and Agriculture Organization of the United Nations, 2002).
10. Cornell University Astronomy Department. *What is the significance of the Tropic of Cancer, Tropic of Capricorn, Arctic Circle and Antarctic Circle? <*

http://curious.astro.cornell.edu/our-solar-system/the-earth/160-our-solar-system/the-earth/seasons/15-what-is-the-significance-of-the-tropic-of-cancer-tropic-of-capricorn-arctic-circle-and-antarctic-circle-beginner> (2003).

11. Laskar, J., Joutel, F. & Robutel, P. Stabilization of the Earth's obliquity by the Moon. *Nature* **361**, 615–617 (1993).
12. ESRI. *ArcGIS Desktop*. (Environmental Systems Research Institute, Redlands, CA, USA, 2010).
13. Bivand, R., Keitt, T. & Rowlingson, B. *rgdal: Bindings for the Geospatial Data Abstraction Library*. R package version 0.8-16. <http://CRAN.R-project.org/package=rgdal> (2014).
14. Olson, D. M. *et al.* Terrestrial Ecoregions of the World: A New Map of Life on Earth A new global map of terrestrial ecoregions provides an innovative tool for conserving biodiversity. *BioScience* **51**, 933–938 (2001).
15. R Core Team. *R: A language and environment for statistical computing.* R Foundation for Statistical Computing, Vienna, Austria. Version 3.1.1. <http://www.R-project.org/> (2014).
16. Bates, D., Maechler, M., Bolker, B. & Walker, S. *lme4: Linear mixed-effects models using Eigen and S4*. R package version 1.1-7. <http://CRAN.R-project.org/package=lme4> (2014).
17. Nieuwenhuis, R., te Grotenhui, M. & Pelzer, B. Influence.ME: Tools for Detecting Influential Data in Mixed Effects Models. *R Journ*. 4, 38–47 (2012).
18. Nakagawa, S. & Schielzeth, H. A general and simple method for obtaining R2 from generalized linear mixed-effects models. *Methods Ecol. Evol.* **4**, 133–142 (2013).
19. Juffe-Bignoli, D. *et al. Protected Planet Report 2014* <http://www.unep-wcmc.org/protected-planet-report-2014> (UNEP-WCMC, 2014).
20. Hoskins A.J. *et al.* Downscaling land-use data to provide global 30" estimates of five land-use classes. *Ecol. Evol.* **6**, 3040-3055 (2016).
21. Hurtt, G. C. *et al.* Harmonization of land-use scenarios for the period 1500–2100: 600 years of global gridded annual land-use transitions, wood harvest, and resulting secondary lands. *Clim. Change* **109**, 117–161 (2011).
22. Mu, Q., Zhao, M. & Running, S. W. Improvements to a MODIS global terrestrial evapotranspiration algorithm. *Remote Sens. Environ.* **115**, 1781–1800 (2011).

23. Hijmans, R. J., Cameron, S. E., Parra, J. L., Jones, P. G. & Jarvis, A. Very high resolution interpolated climate surfaces for global land areas. *Int. J. Climatol.* **25**, 1965–1978 (2005).
24. Reuter, H. I. & Hengl, T. Worldgrids - a public repository of global soil covariates. In: *Digital Soil Assessments and Beyond: Proceedings of the 5th Global Workshop on Digital Soil Mapping 2012, Sydney Australia*. (Taylor & Francis Group, 2012).
25. Hengl, T. *et al.* SoilGrids1km — Global Soil Information Based on Automated Mapping. *PLoS ONE* **9**, e105992 (2014).
26. Uchida, H. & Nelson, A. Agglomeration Index: Towards a new measure of urban concentration in Beall, J., Guha-Khasnobis, B. & Kanbur, R. (Eds) *Urbanization and Development: Multidisciplinary Perspectives* (Oxford University Press, 2011).
27. Center for International Earth Science Information Network, Columbia University, (CIESIN), International Food Policy Research Institute (IFPRI), The World Bank & Centro Internacional de Agricultura Tropical (CIAT). *Global Rural-Urban Mapping Project, Version 1* (GRUMPv1): Population Density Grid. <http://dx.doi.org/10.7927/H4R20Z93> (2011).
28. Tuanmu, M.-N. & Jetz, W. A global 1-km consensus land-cover product for biodiversity and ecosystem modelling. *Glob. Ecol. Biogeogr.* **23**, 1031–1045 (2014).
29. Lehner, B. & Döll, P. Development and validation of a global database of lakes, reservoirs and wetlands. *J. Hydrol.* **296**, 1–22 (2004).
30. Jennions, M. D. & Møller, A. P. Publication bias in ecology and evolution: an empirical assessment using the method. *Biol. Rev.* **77**, 211–222 (2002).
